# A Multifunctional Potent Lewis Acid for In Situ Formation of Poly‐Dioxolane Electrolytes Toward High‐Performance Quasi‐Solid State Lithium Metal Batteries

**DOI:** 10.1002/advs.202519181

**Published:** 2025-11-30

**Authors:** Jaehyeong Yu, Seochan Hong, Minseon Park, Minguk Kwak, Subin Kim, Jaehyun Heo, Won Bae Kim

**Affiliations:** ^1^ Department of Battery Engineering Graduate Institute of Ferrous & Eco Materials Technology (GIFT) Pohang University of Science and Technology (POSTECH) 77 Cheongam‐ro, Nam‐gu, Gyeongbuk‐do Pohang‐si 37673 Republic of Korea; ^2^ Department of Chemical Engineering Pohang University of Science and Technology (POSTECH) 77 Cheongam‐ro, Nam‐gu Pohang‐si Gyeongsangbuk‐do 37673 Republic of Korea

**Keywords:** gel polymer electrolyte, hybrid solid electrolyte interfaces, in situ polymerization, Li metal anode, lithium‐ion battery

## Abstract

Quasi‐solid‐state polymer electrolytes represent a promising strategy for Li metal batteries (LMBs) with superior safety and energy density. However, Li dendrite formation and unstable interfaces significantly hinder their practical application. Here, an AlCl_3_‐initiated gel polymer electrolyte (AGPE) is developed via in situ ring‐opening polymerization of 1,3‐dioxolane (DOL) to directly generate poly(1,3‐dioxolane) (PDOL) electrolyte in battery cells. AlCl_3_ acts both as polymerization initiator and a multifunctional additive, enhancing polymer network stability and facilitating selective Li^+^ transport through an AlCl_3_‐mediated multi‐coordination framework. Additionally, AlCl_3_ spontaneously generates a hybrid SEI layer composed of LiF, LiCl, and LiAl, significantly enhancing interfacial stability and suppressing dendritic growth. Consequently, the AGPE achieves excellent ionic conductivity (≈5.0 mS cm^−1^ at room temperature) and an outstanding Li^+^ transference number (t_Li+_ = 0.75). Li||LiFePO_4_ full cells employing AGPE exhibit superior electrochemical stability, retaining 92.7% capacity after 280 cycles at 0.5 C and delivering a high capacity of 118.2 mAh g^−1^ at 5 C. These results highlight AGPE as an attractive quasi‐solid electrolyte, demonstrating substantial promise for safe and high‐performance next‐generation LMBs.

## Graphical abstract

Conventional polymer electrolytes based on poly‐dioxolane (PDOL) suffer from low ion mobility and unstable interfaces. Here, AlCl_3_‐initiated in situ polymerized gel PDOL electrolytes (AGPE) form a facile Li^+^ conduction path and hybrid SEI layers, thus enabling high Li^+^ conductivity and uniform Li deposition. These findings establish AlCl_3_‐initiated AGPE as a promising platform for the development of high‐performance but safe LMBs.

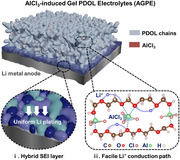

## 1 Introduction

Li metal batteries (LMBs) have garnered considerable interest as promising next‐generation energy storage systems owing to their exceptionally high theoretical specific capacity (3860 mAh g^−1^) and highly negative standard reduction potential (−3.04 V vs SHE).^[^ ^1^, ^2^, ^3^ ^]^ Despite these attractive attributes, the practical deployment of LMBs is significantly impeded by persistent issues such as dendrite growth, unstable solid electrolyte interphase (SEI) layers, and low Coulombic efficiency.^[^ ^4^ ^]^ These challenges are further exacerbated by interactions with electrolytes, especially the widely used carbonate‐based liquid electrolytes (LE), which have high volatility and flammability. Upon contact with Li metal, these electrolytes undergo a direct parasitic reaction, wherein they persistently react with the Li metal anode (LMA) to deplete the electrolyte and build up the electrode/electrolyte interphases. These changes trigger an uncontrolled escalation of cell resistance, ultimately causing early battery failure.^[^ ^5^, ^6^ ^]^ Furthermore, under prolonged or elevated temperature operation, electrolyte decomposition is frequently observed, which deteriorates cell stability and lifespan. Thus, modifying existing electrolytes or developing novel electrolyte systems is urgently required.^[^ ^7^, ^8^ ^]^

To fundamentally address these safety concerns and enhance cell stability, solid‐state electrolytes have emerged as promising alternatives which can effectively overcome flammability and leakage issues associated with LEs, while simultaneously suppressing dendrite formation. Solid‐state electrolytes are broadly divided into inorganic,^[^ ^9^ ^]^ polymer,^[^ ^10^ ^]^ and composite types.^[^ ^11^ ^]^ Among these, polymer electrolytes exhibit significant potential due to their flexible mechanical properties, ease of processing, and excellent interfacial compatibility with electrodes.^[^ ^12^ ^]^ Polyethylene oxide (PEO), a representative polymer electrolyte, offers advantages such as high Li salt solubility and excellent film formability. Nevertheless, its practical application in LMBs is severely restricted by intrinsic drawbacks, including high crystallinity, low room‐temperature ionic conductivity, and insufficient interfacial stability with electrodes.^[^ ^13^ ^]^ To further mitigate interfacial impedance while retaining the processing benefits of polymers, gel polymer electrolytes (GPEs) have been widely adopted as hybrids that combine the merits of solids and liquids. GPEs, in which LEs are physically immobilized within polymer networks, offer both enhanced flexibility and improved ionic conductivity. However, when GPE layers are prepared ex situ, microscopic gaps and compositional gradients can remain at the electrode interface. These defects give rise to additional interfacial resistance, which becomes more pronounced under practical loadings and at high currents. Accordingly, advanced processing routes that enhance interfacial conformity and compositional uniformity have attracted attention.^[^ ^14^ ^]^

In this context, in situ polymerization, which forms the polymer network directly inside assembled battery cells, has emerged as a compelling strategy.^[^ ^15^ ^]^ This approach enhances electrode‐electrolyte interfacial compatibility, ensures compositional uniformity, and simplifies the processing of cells. As a result, it is particularly suitable for high‐energy‐density LMB cell designs. Among these strategies, ring‐opening polymerization of 1,3‐dioxolane (DOL) using Lewis acid initiators to form poly(1,3‐dioxolane) (PDOL) has yielded promising results.^[^ ^16^ ^]^ Early investigations identified Al(OTf)_3_ as an initiator capable of driving DOL ring‐opening polymerization at very low concentrations, and subsequent studies expanded the initiator species including SnCl_4_,^[^ ^17^ ^]^ SnF_2_,^[^ ^18^ ^]^ Sc(OTf)_3_,^[^ ^19^ ^]^ and Mg(OTf)_2_^[^ ^20^ ^]^ to advance PDOL formation and systematically tune network properties. Additionally, the deliberate addition of selected metal cations has attracted increasing attention for inducing Li‐alloy‐based SEI layers, thereby facilitating more uniform Li deposition. Notably, Wu et al. demonstrated that SbF_3_ could initiate in situ polymerization, which led to the formation of LiSb alloy‐enriched SEI layers. These layers played a crucial role in effectively suppressing Li dendrite growth.^[^ ^21^ ^]^ Yang et al. similarly developed stable hybrid SEI layers using InCl_3_‐initiated DOL polymerization.^[^ ^22^ ^]^ However, the limited Lewis acidity of conventional initiators constrains control over the network structure and Li^+^ transport, and stronger Lewis acids are therefore of interest to further enhance ionic conductivity and Li^+^ transference number (t_Li+_).

Complementary to the pursuit of stronger Lewis acids as initiators, recent approaches have combined initiators with functional additives to overcome these limitations. For instance, Zhao et al. utilized AlF_3_ to simultaneously induce in situ ring‐opening polymerization of ether‐based electrolytes and to act as a Lewis acid catalyst and fluorinated cathode electrolyte interphase (CEI) former.^[^ ^23^ ^]^ This design successfully suppressed electrolyte and current collector decomposition at high‐voltage NCM cathodes, achieving a high specific capacity of 153 mAh g^−1^ and stable operation under a high areal capacity of 3.0 mAh cm^−2^. However, this study primarily targeted high‐voltage cathodes and lacked detailed analysis regarding interfacial reactions with LMA and the underlying ion transport mechanisms. Meanwhile, Ma et al., introduced nanoscale LiF and LiBF_4_ into a DOL‐based electrolyte, which increased the polymerization degree of PDOL to 83.19%.^[^ ^24^ ^]^ The incorporation of LiF‐rich SEI and CEI layers led to enhanced interfacial stability and extended cycle life. Nonetheless, the low ambient‐temperature ionic conductivity and limited t_Li+_ remain significant barriers to the realization of high‐power cell applications. In contrast, Hu's group demonstrated a fluorination strategy by adding AlF_3_ into a PEO‐based electrolyte instead of PDOL.^[^ ^25^ ^]^ This approach increased the t_Li+_ to 0.67 and promoted the formation of a conductive SEI at the Li metal interface, enabling over 900 cycles of stable operation. Their work highlights that Lewis acidic species, when employed as functional additives, can play a crucial role in tuning the electrolyte structure and in stabilizing interfacial properties.

**Scheme 1:**
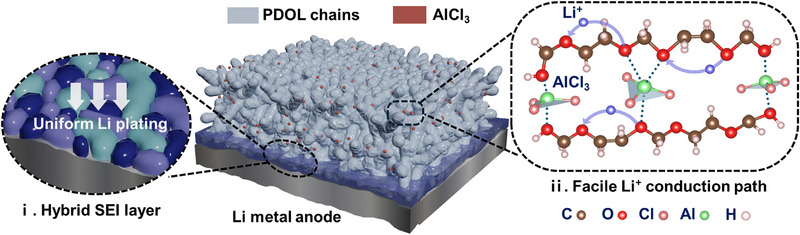
Proposed multi‐functional roles of AlCl_3_ in the PDOL electrolyte for LMBs.

Inspired by these studies, this research presents a novel strategy employing aluminium chloride (AlCl_3_), a strong Lewis acid,^[^ ^26^, ^27^ ^]^ as an initiator for in situ ring‐opening polymerization of DOL, forming AlCl_3_‐induced gel PDOL electrolytes (AGPE) directly within LMB. Notably, AlCl_3_ functions as a Lewis acid initiator that simultaneously acts as a multifunctional additive, even in trace amounts, which reinforces the stability of the polymer network and facilitates Li^+^ transport through multiple coordination interactions with ether groups. The proposed mechanism, depicted in **Scheme** **1**, emphasizes selective Li^+^ pathways and uniform Li deposition facilitated by AlCl_3_‐PDOL interactions. Consequently, the synthesized AGPE exhibits high ionic conductivity (≈5.0 mS cm^−1^ at room temperature) and a notably high Li^+^ transference number (t_Li+_ = 0.75), effectively enhancing Li^+^ transport efficiency. Additionally, AlCl_3_ and LiTFSI undergo interfacial reactions with the highly reactive LMA, leading to the spontaneous formation of a hybrid SEI layer consisting of LiF/LiCl/LiAl, promoting Li^+^ adsorption and diffusion through the formation of a uniform stable interfacial structure. Application in Li||LFP full cells exhibited excellent electrochemical stability under active‐material loading of 5.4 mg cm^−2^, retaining 92.7% of its initial capacity after 280 cycles at 0.5 C. It also demonstrated outstanding high‐rate performance, delivering 118.2 mAh g^−1^ at 5 C and markedly outperforming comparable systems. Collectively, these results substantiate the AGPE as a practical electrolyte platform for next‐generation LMBs.

## 2 Results and Discussion

### 2.1 Characterizations of Prepared AGPE Materials

**Figure 1:**
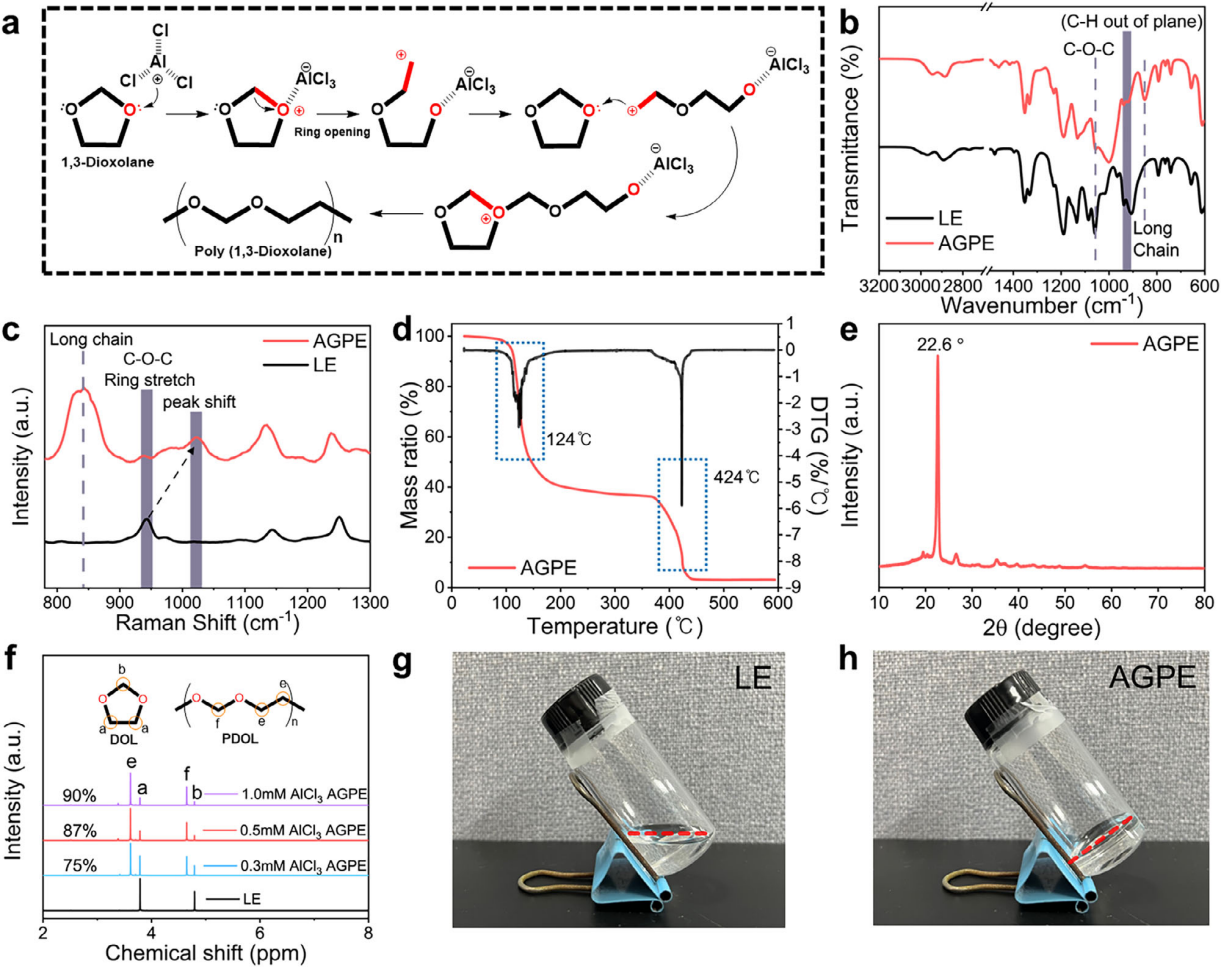
a) Reaction steps via the ring‐opening polymerization of DOL in the presence of Lewis acid of AlCl_3._ b) FTIR spectra of LE and AGPE. c) Raman spectra of LE and AGPE. d) TGA and DTG curves of AGPE. e) XRD patterns of AGPE. f) NMR spectra of LE and AGPE formed at different AlCl_3_ concentrations. Optic images of the g) LE and h) AGPE at room temperature after 24 h from the synthesis.

The potential effects of AlCl_3_ use for theproduction of quasi‐solid‐state polymer electrolytes were first investigated through its ability as the initiator for the ring‐opening polymerization of DOL, thereby assessing its effectiveness in synthesizing polymer gel electrolytes. To clarify the underlying polymerization procedure, **Figure** **1**a illustrates that the ring‐opening polymerization of DOL is initiated through nucleophilic attack by the positively charged Al species from AlCl_3_, a strong Lewis acid which can strongly attract the lone electron pairs on the oxygen atoms of the DOL ring. This process breaks the cyclic C─O bonds, generating highly reactive oxonium intermediates (i.e., carbonium‐like species) through cleavage of the cyclic C─O bonds. These electrophilic intermediates subsequently initiate chain growth by sequentially attacking the oxygen atoms of other DOL monomers, thereby propagating the ring‐opening polymerization process.^[^ ^28^ ^]^

Fourier transform infrared spectrometer (FTIR) and Raman spectroscopy analyses revealed structural changes in accordance with this phase transition. The FTIR spectra (Figure 1b) represented characteristic peaks for DOL before polymerization at 900–950 cm^−1^ (C─H out‐of‐plane vibrations) and 1058 cm^−1^ (C─O─C). After polymerization of DOL forming into PDOL, these peaks were diminished with a shift to ≈1000 cm^−1^, while a new peak at 852 cm^−1^ corresponding to O─C─O chain vibrations appeared, indicating that the successful ring‐opening polymerization was achieved.^[^ ^17^, ^19^ ^]^ Further, as the initiator concentration increases, the long‐chain peaks intensify while the DOL‐specific bands weaken (Figure S1, Supporting Information), which is consistent with the observation of more extensive polymerization leading to smaller residual DOL content. Raman spectra also supported the polymerization reaction, as shown in Figure 1c. The characteristic C─O─C stretching vibration peak of DOL, originally observed in the range of 940–960 cm^−1^, undergoes a notable shift to a higher wavenumber region ≈1022 cm^−1^ upon polymerization. This shift is indicative of a change in the molecular environment of the ether ring, reflecting the ring‐opening polymerization of DOL. Additionally, a new peak appears ≈840 cm^−1^, corresponding to the C─O and C─H vibrational modes associated with the PDOL backbone. As shown in Figure S2 (Supporting Information), the intensity of this band increases with higher AlCl_3_ concentrations, in agreement with the FTIR observations discussed above for the effect of the increased initiator concentrations.^[^ ^29^ ^]^ The concurrent appearance of this new vibrational signal and the blue shift of the original ether peak serve as direct spectroscopic evidence for the successful formation of the polymeric network. These spectral changes indicate that the ring‐opening polymerization proceeds efficiently, leading to the conversion of monomeric DOL into a crosslinked PDOL structure and validating the occurrence and completion of the polymerization process.

The thermal stability of the synthesized AGPE was assessed using thermogravimetric analysis (TGA) and derivative thermogravimetry (DTG) (Figure 1d). Pure DOL is known to volatilize at ≈75 °C, whereas the polymerized PDOL in this work exhibited a substantial weight loss ≈124 °C. A further decomposition ≈424 °C should be attributed to LiTFSI. Higher AlCl_3_ concentrations seem to improve thermal stability by reducing unpolymerized DOL content,^[^ ^18^, ^30^ ^]^ as supported by Figure S3 (Supporting Information). X‐ray diffraction (XRD) was performed to assess the crystallinity of PDOL chains (Figure 1e). Distinct diffraction peaks at 22.6°, obtained from PDOL powder washed thoroughly with deionized water by the centrifuge method (Figure S4, Supporting Information), indicated the existence of crystalline domains in the polymerized PDOL.^[^ ^31^ ^] 1^H NMR spectroscopy (Figure 1f) could quantify the polymerization conversion degree, where DOL peaks at 3.78 ppm (─CH_2_CH_2_O─) and 4.78 ppm (─CH_2_OCH_2_─) shifted to 3.61 ppm (─OCH_2_CH_2_O─) and 4.64 ppm (─OCH_2_O─), respectively. The conversion fraction of the DOL monomer (C_m_) was calculated using the integrated peak areas according to Equation (1):^[^ ^19^ ^]^

(1) $$C_{m}=\frac{e}{a+e}$$

where *e* represents the integrated area of the ^1^H NMR peak assigned to the methylene protons (─OCH_2_CH_2_O─) in the PDOL repeating units, and a corresponds to the integrated area of the peak assigned to the methylene protons (─CH_2_CH_2_O─) in the unreacted DOL monomer, as labelled in Figure 1f. The C_m_ value was increased as the AlCl_3_ concentration increased, reaching 75% at 0.3 mm, 87% at 0.5 mm, and 90% at 1.0 mm, which can be attributed to the increased availability of initiating species that accelerate the ring‐opening polymerization of DOL. This result highlights that efficient polymerization can be achieved even at extremely low initiator concentrations.^[^ ^32^ ^]^ To compare the molecular weights of PDOL at different initiator concentrations, MALDI‐TOF analysis was performed. As shown in Figure S5 (Supporting Information), the distribution shifts toward lower molecular weights with increasing initiator concentration. This phenomenon can be explained by the fact that a higher initiator content could induce multiple simultaneous ring‐opening polymerization events within the limited amount of DOL monomer, resulting in the formation of shorter polymer chains.^[^ ^32^, ^33^ ^]^

Figure 1g,h depict the photographs of the LE (DOL/LiTFSI) and the AGPE (PDOL/LiTFSI) that were prepared with the AlCl_3_ initiator, respectively, which were taken 24 h after the synthesis at room temperature. A successful AGPE formation seemed to be achieved, as the transparent liquid DOL/LiTFSI mixture transformed into a gel state with the addition of only 0.5 mm AlCl_3_. A notable observation arises from the image presented in Figure S6 (Supporting Information) in that the polymerization does not occur even at concentrations as high as 10 mm AlCl_3_ without the addition of LiTFSI. Based on this result, it is believed that the interaction between the TFSI^−^ anion of LiTFSI and AlCl_3_ plays a critical role in enhancing the solubility of AlCl_3_ in DOL.^[^ ^18^ ^]^ This finding suggests that LiTFSI not only functions as a Li salt in the electrolyte but also contributes to the activation of the initiator, thereby influencing the initiation and progression of the polymerization reaction.

### 2.2 Mechanistic Investigation of Ion Conduction on AGPE

**Figure 2:**
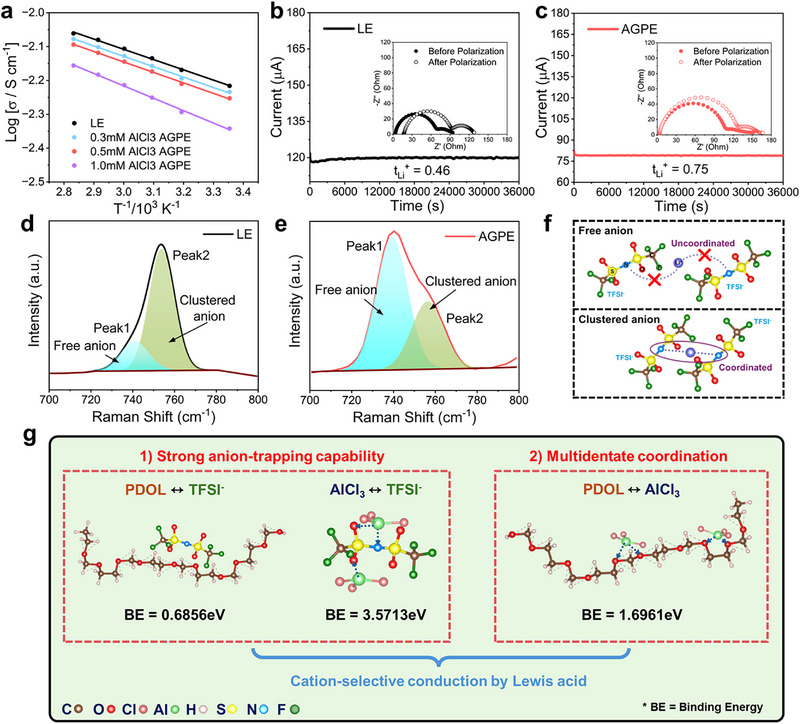
a) Arrhenius plots of the conductivity of LE and AGPE formed at different AlCl_3_ concentrations. Current‐time curves following DC polarization of 10 mV for Li||Li symmetric cells with electrolytes of b) LE and c) AGPE. Raman spectra of d) LE and e) AGPE in the wavenumber range of 700–800 cm^−1^ for the comparison of dissociation effect of AlCl_3_ on LiTFSI. f) Schematic illustration of the coordination states between Li⁺ and free or clustered anions. g) Schematic illustration of the ionic conduction pathways governed by the binding energies between PDOL, TFSI^−^ and AlCl_3_.

**Figures** **2**a and S7 (Supporting Information) compare the temperature‐dependent ionic conductivity of the AGPEs synthesized by in situ polymerization and the LE. Measurements were conducted using stainless steel (SS) || SS cells via electrochemical impedance spectroscopy (EIS) (Figure S8a–d, Supporting Information). Across the entire temperature range, the LE exhibited higher ionic conductivity than the those of AGPEs. The ionic conductivity of AGPE was gradually decreased with increasing AlCl_3_ concentration, attributed primarily to the decreasing proportion of unpolymerized DOL molecules, which could promote the ion mobility. Nonetheless, the AGPE still showed an excellent ionic conductivity (≈5 mS cm^−1^ at room temperature), suggesting its effective ion migration ability is facilitated by the polymer network. Although the overall ionic mobility may be high, the decisive parameter is the t_Li+_, which represents the fraction of the total current conveyed by Li^+^. To quantify t_Li+_, we have performed chronoamperometry in a Li||Li symmetric cell under a 10 mV DC bias, complemented by AC impedance measurements before and after polarization (Figure 2b,c). The transference number was calculated according to the Vincent‐Evans Equation (2):

(2) $$t_{Li^{+}}=\frac{I_{SS}\left(\Delta V-I_{0}R_{0}\right)}{I_{0}\left(\Delta V-I_{SS}R_{SS}\right)}$$

where ΔV is the applied bias of 10 mV, I_0_ and I_SS_ are the initial and steady‐state currents, and R_0_ and R_SS_ are the cell resistances before and after DC polarization, respectively. The AGPE containing 0.5 mm AlCl_3_ exhibits the transference number t_Li+_ of 0.75, which is markedly higher than that of the LE (0.46). This enhancement is attributed to AlCl_3_ functioning as a Lewis acid that immobilizes TFSI^−^ anions, thereby reducing anionic contribution to charge transport and increasing the relative share carried by Li^+^.

To further elucidate the molecular origins of this improvement on t_Li+_, additional Raman spectroscopy (Figure S9, Supporting Information; Figure 2d,e) was conducted to examine the TFSI^−^ bonding states in lower Raman shift regions. Peaks at 740–747 cm^−1^ correspond to free TFSI^−^, and 747–750 cm^−1^ to ion clusters.^[^ ^25^, ^34^, ^35^ ^]^ The AGPE showed an increased proportion of free TFSI^−^ as compared to the LE, suggesting that AlCl_3_ promotes dissociation of Li⁺–anion pairs. This increased availability of free TFSI^−^ reduces Li^+^–anion association, thereby increasing the fraction of Li^+^ participating in conduction and resulting in a higher Li‐ion transference number, as visually schematized in Figure 2f. A complementary ^7^Li NMR analysis (Figure S10, Supporting Information) further supports this observation, showing an upfield chemical shift for the AGPE compared with the liquid electrolyte. This shift reflects a weaker solvation environment and reduced Li^+^–TFSI^−^ coordination strength, implying that a larger fraction of Li^+^ exists in a more mobile and weakly coordinated state.^[^ ^33^, ^36^ ^]^ These results corroborate the Raman findings, supporting that the Lewis‐acidic AlCl_3_ additive effectively disrupts ion pairing and facilitates enhanced Li^+^ transport in the AGPE.

Density functional theory (DFT) calculations (Figure 2g) provided further atomic‐scale insights into the ion transport mechanism by analyzing the binding interactions among PDOL, AlCl_3_, and TFSI^−^. In the AGPE system, the binding energy between the ether oxygen atoms in PDOL and the TFSI^−^ anion is merely 0.68 eV, indicative of weak dipole–ion interactions. This relatively low affinity indicates that TFSI^−^ anions preferentially interact with AlCl_3_ species, rather than with the PDOL chains. The central Al atom, characterized by its strong Lewis acidity with a high positive charge, can interact intensively with the electron‐rich TFSI^−^ anions. This interaction arises from both electrostatic attraction and partial coordination between Al and fluorine or oxygen atoms within the TFSI^−^ structure. As a result, the binding energy increases markedly to 3.57 eV, representing at least fivefold enhancement as compared to the PDOL–TFSI^−^ binding energy. By preferentially adsorbing onto the central Al atoms, the TFSI^−^ anions are effectively dissociated from Li^+^, which results in the release of free Li^+^ and promotes ionic transport.^[^ ^25^ ^]^ Simultaneously, PDOL chains exhibit a binding energy of 1.69 eV with AlCl_3_, consistent with multi‐dentate coordination between ether oxygens and the central Al atom. This assignment is supported by DFT analysis, which yields an Al─O separation of 1.935 Å and a computed bond order of 0.399, representing characteristics of a genuine coordination interaction rather than a weak non‐bonded contact. Such bridge‐like interactions tether AlCl_3_ to the polymer backbone and create a percolating network of Lewis‐acid sites that guide desolvated Li^+^ along well‐defined conduits. Consequently, the coexistence of i) strong anion sequestration via Al–TFSI^−^ interactions and ii) robust PDOL–AlCl_3_ coordination provides a dual synergistic mechanism. Specifically, this dual effect suppresses anionic drag by immobilizing TFSI^−^, while simultaneously furnishing continuous, cation‐selective migration pathways for Li^+^. Thus, the dual‐mechanism model provides a mechanistic explanation for the exceptionally high t_Li+_ as experimentally observed in the AGPE. Taken together, these findings offer compelling atomic‐level evidence supporting our proposed AlCl_3_‐mediated multi‐coordination framework, clearly highlighting its pivotal role in decoupling Li^+^ from TFSI^−^ and establishing efficient ion‐transport highways within the polymer gel matrix.

### 2.3 Electrochemical Stability of AGPE

**Figure 3:**
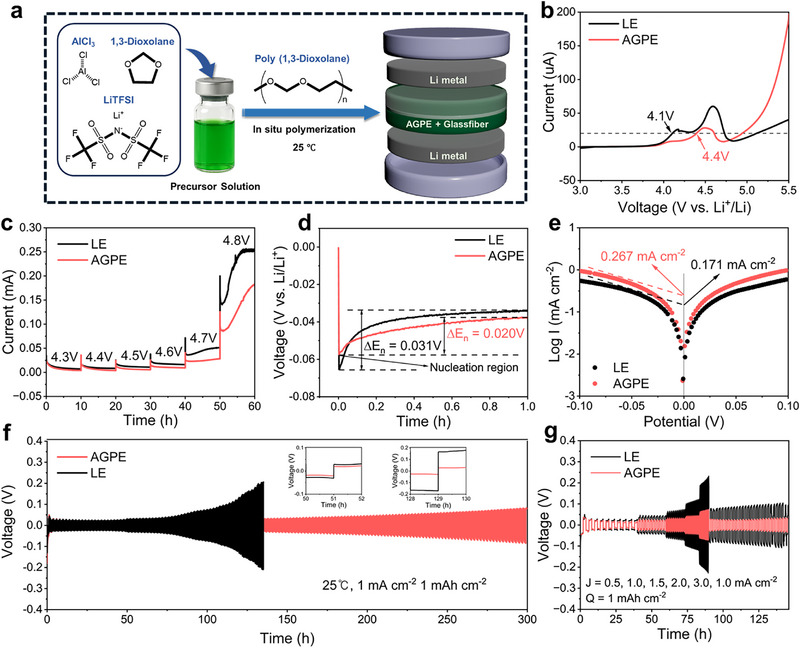
a) Schematic illustration of coin cell configuration of Li||Li symmetric cell fabricated via in situ polymerization of electrolyte. b) linear sweep voltammograms of LE and AGPE at a scan rate of 1 mV s^−1^ using stainless steel as the working electrode and Li foils as the counter electrode and reference electrode. c) Electrochemical floating analysis of cells using NCM811 cathodes for AGPE. d) Nucleation overpotential of Li||Li symmetric cells assembled with LE and AGPE. e) Tafel plots for Li plating/stripping of the Li||Li symmetric cells with LE and AGPE. f) Li plating/stripping profiles of Li||Li symmetric cells assembled with LE and AGPE under 1 mA cm^−2^. g) Rate performance of Li||Li symmetric cells assembled with LE and AGPE at various current densities.

The electrochemical characteristics of AGPE were evaluated using Li||Li symmetric cells. **Figure** **3**a illustrates the in situ polymerization process of AGPE within cells, where a precursor solution containing AlCl_3_, DOL, and LiTFSI was injected between Li electrodes using glass fiber separators (Figure S11, Supporting Information).^[^ ^37^ ^]^ Cells fabricated under 0.5 mm AlCl_3_ and rested for 24 h were selected as the optimal condition on the basis of Li||Li symmetric cell through screening initiator concentration and rest time, showing the lowest polarization and stable voltage maintenance (Figures S12 and S13, Supporting Information). Linear sweep voltammetry (LSV) analysis (Figure 3b) demonstrated that AGPE exhibited an electrochemical stability window (ESW) of ≈4.4 V, significantly surpassing that of LE (≈4.1 V). The oxidative current onset was defined at 20 µA, a threshold commonly adopted in literature to reliably distinguish true Faradaic reactions from background capacitive currents.^[^ ^38^ ^]^ DFT calculations (Figure S14, Supporting Information) support that the AlCl_3_, possessing a low highest occupied molecular orbital (HOMO) level, forms multiple coordination bonds with the ether oxygens of PDOL and TFSI^−^ anions, thereby enhancing the oxidative stability of the electrolyte and extending its ESW.^[^ ^22^, ^39^ ^]^ Furthermore, LSV measurements conducted with varying AlCl_3_ concentrations (Figure S15, Supporting Information) showed that the electrochemical stability gradually improved with increasing polymerization degree but reached a saturation point beyond ≈0.5 mm, suggesting that excessive polymerization could provide limited additional benefit to the ESW. Floating tests with NCM 811 cathodes (Figure 3c) further confirmed the superior high‐voltage interfacial stability of AGPE. Notably, the AGPE‐based cell exhibited remarkably low leakage currents, consistently maintained below 30 µA even at elevated potentials up to 4.7 V. In contrast, the cell employing a conventional LE showed higher leakage currents under the same conditions, indicative of undesirable interfacial side reactions and inferior electrochemical stability.^[^ ^40^, ^41^ ^]^ These results clearly demonstrate that the AGPE developed in this work effectively suppresses parasitic oxidation reactions at the cathode–electrolyte interface, thereby enabling stable operation in the high‐voltage regime. Such characteristics underscore the potential of AGPE for application in high‐energy‐density quasi‐solid‐state LMBs, where both interfacial compatibility and voltage tolerance are critical.

As shown in Figure 3d, the cell with AGPE exhibited a low nucleation overpotential of 20 mV, which is notably lower than that of LE (31 mV), indicating that more uniform Li deposition occurred. This low overpotential is attributed to the well‐organized polymer network formed by interactions among AlCl_3_, TFSI^−^, and PDOL, which stabilizes Li^+^ migration pathways and increases the proportion of free ions. Consequently, Li^+^ reaches the interface more efficiently, reducing the energy barrier for nucleation. To quantify the Li^+^ transfer capability, Tafel analysis was performed (Figure 3e). The AGPE exhibited a higher exchange current density of 0.267 mA cm^−2^ compared to 0.171 mA cm^−2^ for the LE, indicating less charge transfer resistance and more favorable pathways for rapid and efficient Li plating/stripping. The long‐term reversibility of Li plating/stripping in AGPE was evaluated using Li||Li symmetric cells under conditions of 1 mA cm^−2^ and 1 mAh cm^−2^ at 25 °C (Figure 3f). Although the LE initially showed lower polarization, it gradually increased after 50 h, reaching a value ≈150 mV higher than that of AGPE after 129 h. This increase is attributed to the growth of a thick SEI and the accumulation of electrically isolated “dead lithium.” In contrast, the cell with AGPE maintained stable cycling for over 300 h, due to the continuous Li^+^ transport channels stabilized by AlCl_3_‐induced multiple coordination bonding, which effectively suppressed interfacial resistance build‐up. In addition, symmetric cell tests conducted at different temperatures (Figure S17, Supporting Information) further verified the robustness of AGPE. At 10 °C, the AGPE showed much lower overpotential and more stable interfacial behavior than the liquid electrolyte, owing to its higher Li^+^ transference number and well‐preserved polymer–Li interface that mitigates ion‐concentration polarization.^[^ ^33^ ^]^ Even at 50 °C, the AGPE maintained stable cycling without degradation or short‐circuiting, attributed to its cross‐linked polymer network and AlCl_3_‐derived hybrid interphase that can prevent thermal decomposition.^[^ ^42^ ^]^ Figure 3g further confirmed that, even under high current densities in rate‐capability tests with Li||Li symmetric cells, AGPE exhibited consistently lower overpotentials than the LE. This performance is attributed to the robust interfacial stability and fast ion conduction enabled by the polymeric network structure and AlCl_3_‐mediated coordination, which can prevent Li dendrite formation and ensure homogeneous Li deposition.

### 2.4 Post Analysis of Li||Li Symmetric Cell

**Figure 4:**
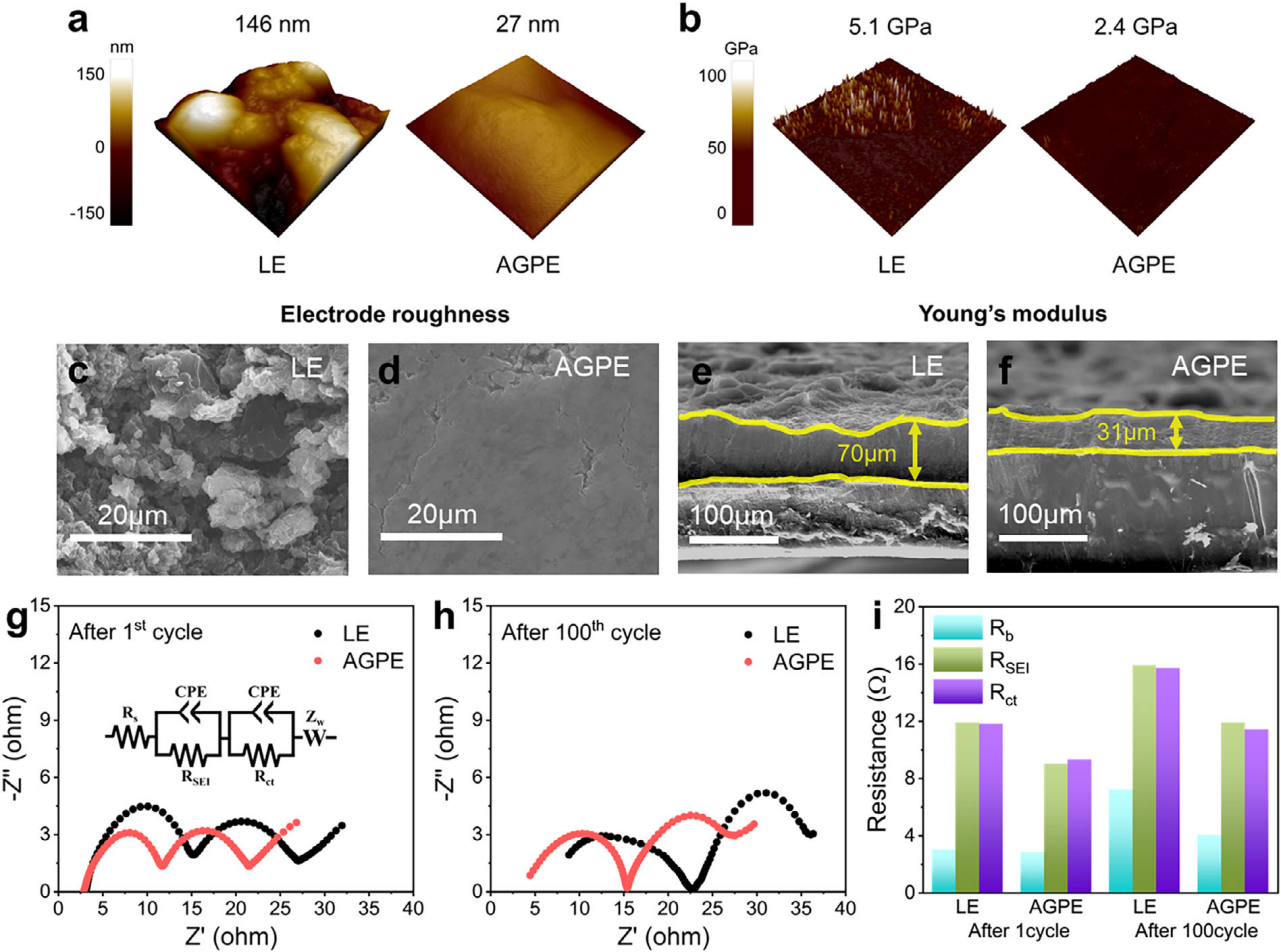
AFM characterizations of the a) roughness, b) Young's modulus mapping of LMA after cycling Li||Li symmetric cells assembled by LE and AGPE. Cross‐view and top‐view SEM images of LMA after cycling Li||Li symmetric cells assembled by c,e) LE and d,f) AGPE. Corresponding Nyquist plots of the EIS measurements of Li||Li symmetric cells assembled by LE and AGPE g) after 1st cycle and h) after 100th cycles and i) the obtained different types of resistance. The inset in Figure 4g is the equivalent circuit.

The exceptional electrochemical stability of AGPE demonstrated in Figure 3 was further validated through various post‐mortem analyses, focusing on the structural and mechanical integrity of the Li metal interface after prolonged cycling. To characterize the LMA surface after 100 h of cycling, atomic force microscopy (AFM) was conducted on Li||Li symmetric cells. As illustrated in **Figure** **4**a, the LE cell exhibited a root‐mean‐square roughness (R_rms_) of 146 nm, whereas the AGPE cell maintained a much smoother surface with R_rms_ of 27 nm, evidencing more uniform Li plating. To elucidate the origin of this improved morphology, Young's modulus was subsequently mapped (Figure 4b), and the LE interface registered 5.1 GPa, while the AGPE interface showed a lower value of 2.4 GPa. An elastically compliant SEI with moderate stiffness can accommodate the volume expansion and contraction of Li, thereby mitigating micro‐cracking and delamination. Conversely, an excessively rigid inorganic layer is brittle and prone to cracking and spallation during Li plating/stripping. Therefore, the intermediate modulus of the AGPE interface underpins its low roughness and superior electrochemical stability.^[^ ^43^ ^]^ Complementarily, AFM revealed a substantially reduced energy dissipation (Figure S18, Supporting Information) for the AGPE cell (0.83 fJ) compared with the LE cell (1.22 fJ), indicating diminished viscoelastic losses and adhesive hysteresis that minimize interfacial reconstruction and electrolyte degradation. Collectively, the combination of low roughness, appropriate modulus, and reduced energy dissipation enables AGPE to retard impedance growth and secure long‐term cycling stability.

Figure 4c–f present scanning electron microscopy (SEM) images comparing the top and cross‐sectional views of Li anodes after long‐term cycling. In the LE cell (Figure 4c), the surface morphology revealed coarse and protruding dendritic structures caused by nonuniform Li deposition. In contrast, the AGPE cell (Figure 4d) displayed a smoother and more uniform deposition layer, consistent with the AFM findings. Cross‐sectional images (Figure 4e,f) further confirmed this trend, where the LE cell exhibited a thick and uneven Li layer, while the AGPE cell retained a thinner and more homogeneous Li morphology. These differences are attributed to the low t_Li+_ and irregular ion flux in the LE system, which leads to excessive Li plating and the accumulation of inactive regions. Conversely, AGPE facilitates controlled deposition through well‐aligned polymer networks that maintain stable ion transport pathways.

Figure 4g–i and Table S1 (Supporting Information) illustrate the interfacial resistance evolution of both AGPE and LE cells, analyzed by EIS after the first and 100th cycles. According to the Nyquist plots (Figure 4g,h), the LE cell showed a pronounced increase in semicircle diameter with cycling, indicating significant rises in bulk resistance (R_b_), SEI resistance (R_SEI_), and charge transfer resistance (R_ct_). Although a slight increase in impedance components was also observed for the AGPE cell, the extent of change was considerably smaller. Quantitative comparison via the bar graph in Figure 4i reveals that AGPE consistently maintained lower values for R_b_, R_SEI_, and R_ct_ than the LE cell, with minimal increases even after 100 cycles. This suggests that AGPE effectively preserves interfacial stability by maintaining aligned Li^+^ pathways within the polymer network, thereby suppressing localized current hotspots and dendrite formation and ensuring long‐term electrochemical reliability.^[^ ^44^ ^]^

### 2.5 AlCl_3_‐Induced Hybrid SEI Layer Formation

**Figure 5:**
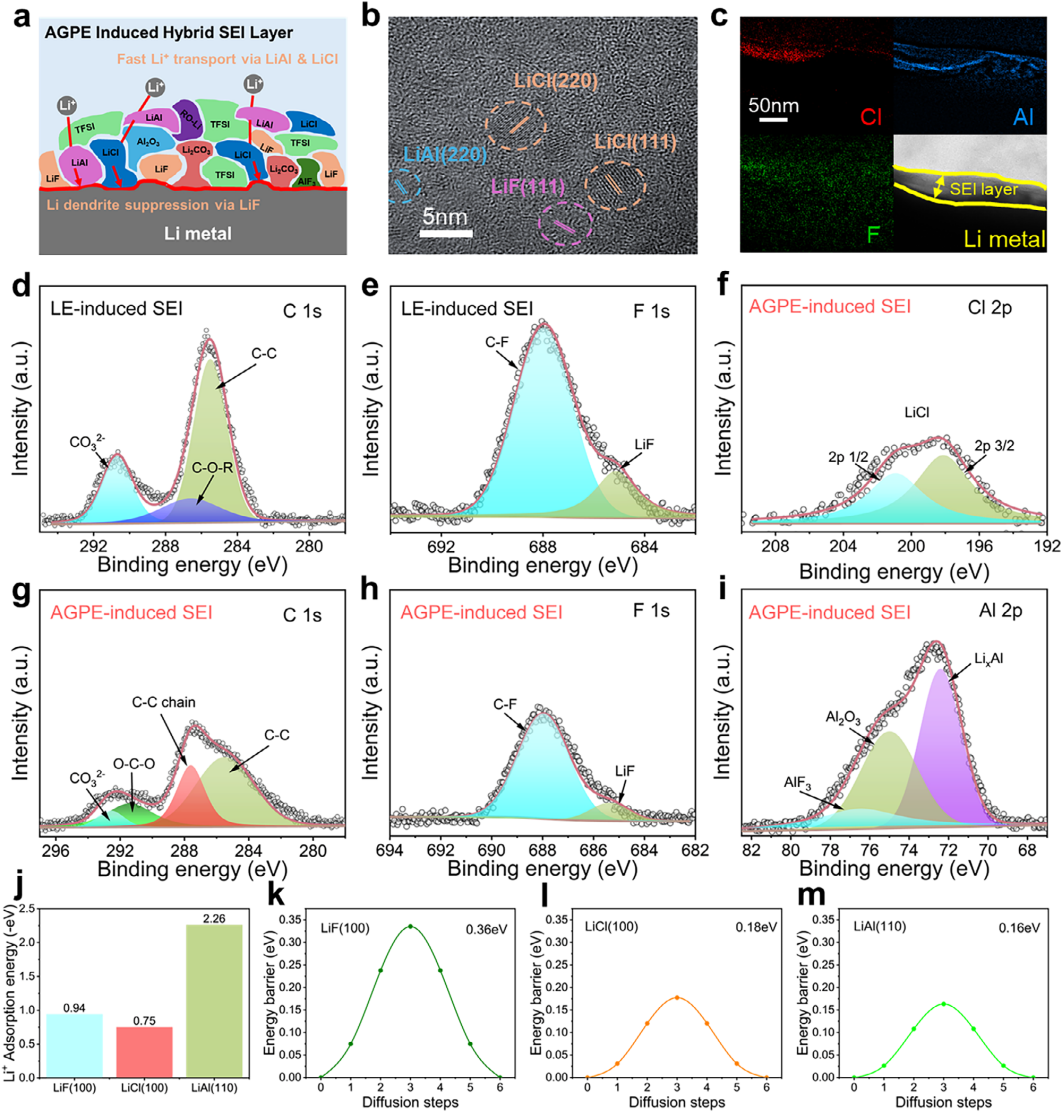
a) Schematic illustration of constituents of hybrid SEI layer for cycled LMA with AGPE after 100 cycles. b) HR‐TEM image of the LiF/LiCl/LiAl hybrid SEI layer. c) The corresponding EELS maps of Cl, Al, and F elements. XPS spectra of d) C 1s, e) F 1s taken from the cycled Li metal surface with LE after 100 cycles. XPS spectra of f) Cl 2p, g) C 1s, h) F 1s, and i) Al 2p taken from the cycled Li metal surface with AGPE after 100 cycles. j) The adsorption energy of Li^+^ on the LiF (100), LiCl (100), and LiAl (110) surfaces. Kinetic energy barriers of Li^+^ diffusion on the k) LiF (100), l) LiCl (100), and m) LiAl (110) surfaces.

LiF is widely recognized for its high Young's modulus and chemical/electrochemical inertness at the Li interface, which contribute to the suppression of Li dendrite growth and the formation of thin, robust SEI layers. Nevertheless, dense LiF is intrinsically a poor Li^+^ conductor and an electronic insulator at ambient conditions. Owing to its high stiffness, LiF‐rich SEI layers tend to be brittle, which raises interfacial resistance and exacerbates polarization at high rates.^[^ ^45^, ^46^ ^]^ In this study, as illustrated in **Figure** **5**a, AlCl_3_ functioning as an initiator for polymerization of electrolyte was also found to directly react with Li metal, leading to the spontaneous formation of a hybrid SEI layer composed of not only LiF but also LiAl and LiCl. These additional inorganic phases (LiAl and LiCl) compensate for the low ionic conductivity of LiF and promote smoother Li^+^ transport, offering synergistic benefits for interfacial stability.^[^ ^22^, ^47^ ^]^

To investigate the structural composition of this hybrid SEI, a Li/AGPE/Cu cell was fabricated and subjected to Li plating at 0.1 mA cm^−2^ up to an areal capacity of 0.5 mAh cm^−2^, followed by observation with high‐resolution transmission electron microscopy (HR‐TEM). As shown in Figure 5b, distinct lattice spacing distances corresponding to LiF, LiCl, and LiAl phases were observed within the SEI layer. Elemental mapping via electron energy loss spectroscopy (EELS, Figure 5c) further represented the homogeneous distribution of Al and Cl, alongside F, throughout the SEI, corroborating the HR‐TEM results.

Additional analysis was carried out via X‐ray photoelectron spectroscopy (XPS) of Li metal retrieved after 30 cycles from a Li||Li symmetric cell cycled at 1 mA cm^−2^ and 1 mAh cm^−2^ (Figure 5d–i). In the C 1s spectrum (Figure 5d), the LE sample displayed prominent peaks corresponding to CO_3_^2−^ and C─O─R species, which are typical products of DOL decomposition, as well as the characteristic C─C bond associated with the cyclic structure of the DOL ring.^[^ ^32^, ^48^ ^]^ These findings indicate that in the liquid electrolyte, DOL molecules are prone to reductive decomposition on the Li surface, forming unstable organic species and carbonate by‐products that contribute to the continuous thickening of the SEI layer. In contrast, the AGPE sample exhibited a significant reduction in the intensities of these decomposition‐related peaks, suggesting the effective suppression of solvent degradation. Instead, new signals assigned to O─C─O and linear C─C chain structures appeared, which are attributed to the formation of polymeric PDOL segments through in situ ring‐opening polymerization. The F 1s spectra (Figure 5e,h) revealed lower intensities for C─F and LiF peaks^[^ ^49^ ^]^ in the AGPE sample, implying that AlCl_3_ suppressed TFSI^−^ mobility and reactivity via coordination, thus minimizing its contribution to SEI formation. Moreover, the Cl 2p^[^ ^50^ ^]^ and Al 2p^[^ ^51^, ^52^ ^]^ spectra (Figure 5f,g) confirmed the presence of LiCl and LiAl, as well as AlF_3_ and Al_2_O_3_, clearly indicating that the hybrid SEI was composed of LiF, LiCl, and LiAl. For the Li 1s XPS spectrum as shown in Figure S19 (Supporting Information), the AGPE sample exhibited not only the characteristic peak of LiF but also distinct signals corresponding to LiCl and LiAl.

To gain mechanistic insights into the electrochemical roles of these components, DFT calculations were performed. The adsorption energy (AE) of Li^+^ on various surfaces was calculated as summarized in Table S2 (Supporting Information). As shown in Figure 5j and Figure S20 (Supporting Information), the adsorption energies were calculated to be −0.94 eV for LiF (100) and −0.75 eV for LiCl (100).^[^ ^22^ ^]^ Notably, LiAl (110) exhibited significantly lower adsorption energies of −2.26 eV, indicating a much stronger interaction with Li^+^. These results suggest that LiAl surfaces possess superior Li^+^ adsorption capability. Furthermore, the Li^+^ diffusion barriers on LiF (100), LiCl (100), and LiAl (110) surfaces were evaluated using the nudged elastic band (NEB) method (Figure 5k–m), and the corresponding diffusion pathways of Li^+^ were illustrated in Figure S21 (Supporting Information). The results showed lower diffusion energy barriers for LiCl (100) (0.18 eV) and LiAl (110) (0.16 eV) compared to LiF (100) (0.36 eV), indicating more facile Li^+^ transport in the former.^[^ ^18^, ^53^ ^]^

Taken together, these experimental and theoretical findings suggest that the AlCl_3_‐initiated hybrid SEI composed of LiF/LiCl/LiAl effectively enhances both Li^+^ adsorption and ion transport at the Li interface. The resulting uniform and compact Li deposition contributes to the superior electrochemical performance of AGPE, thus providing structural and mechanistic justification for its exceptional functionality.

### 2.6 Li||LFP Cell Performance

**Figure 6:**
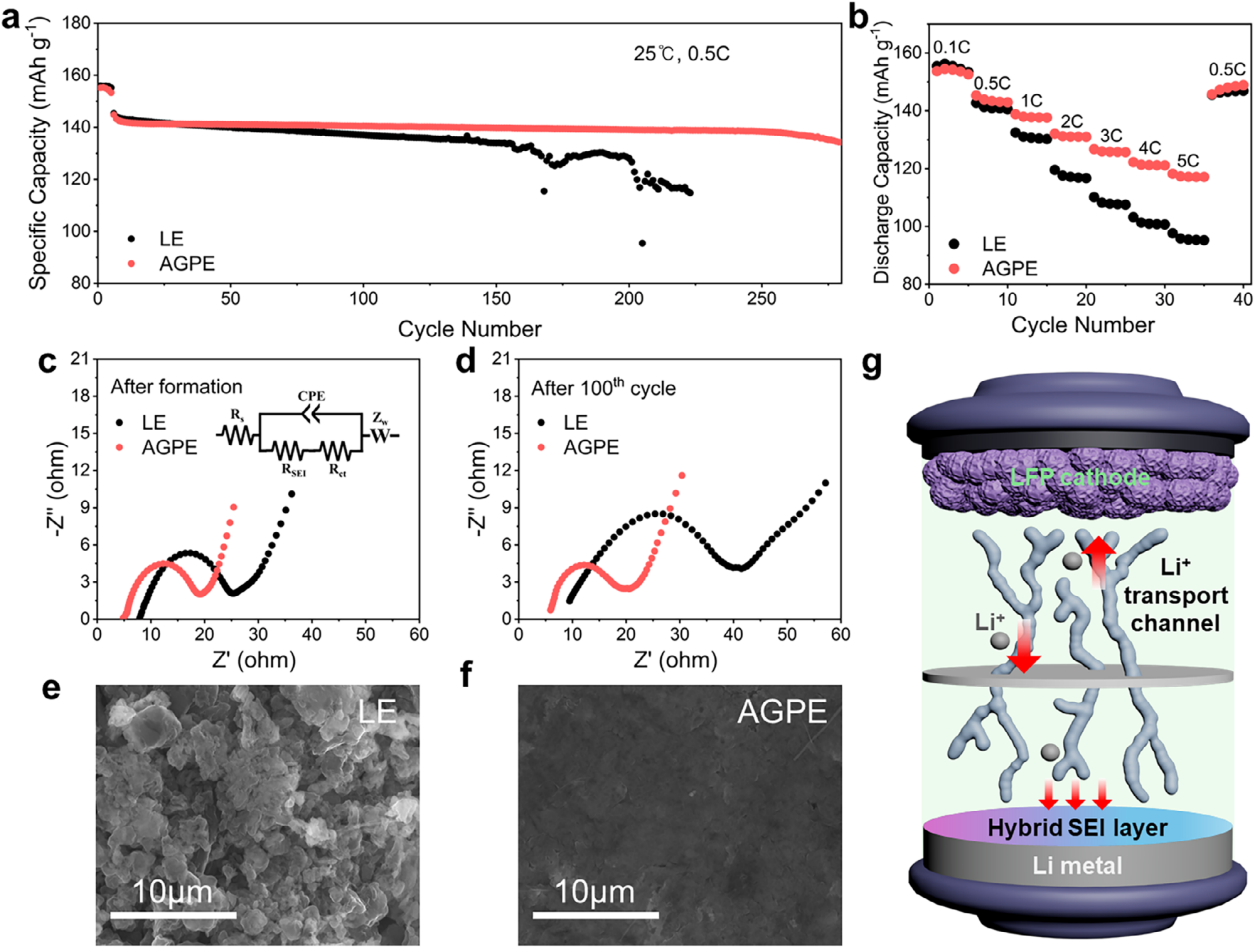
a) Cyclic performance of Li/LE/LFP cells and Li/AGPE/LFP cells at 0.5 C (1 C = 170 mAh g^−1^). b) Rate performance of Li||LFP cells using LE and AGPE as electrolytes. Corresponding Nyquist plots of the EIS measurements of Li||LFP cells assembled by LE and AGPE c) after formation and d) after 100 cycles (inset is the equivalent circuit). Top‐view SEM images of LMA after cycling Li||LFP cells assembled by e) LE and f) AGPE. g) Schematic illustration of an LMB employing an LFP cathode with AGPE.

To evaluate the practical applicability of AGPE in quasi‐solid‐state Li metal batteries (QSLMBs), Li||LFP full cells were assembled using a LiFePO_4_ cathode with an areal loading of 5.4 mg cm^−2^ and Li metal as the anode. **Figure** **6**a and S22 (Supporting Information) compare the long‐term cycling performance of cells employing either a LE or AGPE at 25 °C at 0.5 C. The Li/AGPE/LFP cell delivered an initial discharge capacity of 144.8 mAh g^−1^ after five formation cycles and retained 134.3 mAh g^−1^ after 280 cycles, corresponding to a high‐capacity retention of 92.7%. In contrast, the Li/LE/LFP cell exhibited similar or slightly higher capacity during the first 30 cycles, but then experienced a sharp capacity fade, reaching only 114.7 mAh g^−1^ by the 223rd cycle and a retention rate of 78.9%. Furthermore, the life test conducted at 1 C (Figure S23, Supporting Information) demonstrated that the AGPE maintained a higher capacity retention than the LE, confirming the superior durability and electrochemical robustness of the AGPE even under more demanding operating conditions. These results clearly demonstrate the contribution of AGPE to long‐term stability through continuous Li^+^ transport pathways and the formation of a robust functional SEI. Rate capability tests were further conducted to evaluate the electrochemical performance under various charge/discharge rates (Figure 6b; Figure S24, Supporting Information). At low rates (0.1 and 0.5 C), both LE‐based and AGPE‐based cells showed comparable capacities, consistent with the trends observed in the long‐cycle data. However, as the charge rate increased, the LE cell exhibited a marked capacity drop, while the AGPE cell maintained a relatively stable performance. Notably, under high‐rate conditions (5 C), the AGPE cell delivered a capacity of 118.2 mAh g^−1^, significantly outperforming the LE cell that dropped to 97.6 mAh g^−1^, and thereby highlighting the superior high‐rate capability of the AGPE system. Figure S28 and Table S3 (Supporting Information) compare active material loading, applied C‐rate, and delivered capacity with leading literature reports, and highlight that our AGPE‐based cell exhibits exceptional high‐rate capability.

Cyclic voltammetry (CV) measurements on the Li||LFP cells (Figure S25, Supporting Information) also revealed distinct differences in interfacial stability between the two electrolyte systems. In the LE‐based cell, the reduction peak (≈3.2 V) shifted negatively, and the oxidation peak (≈3.6 V) shifted positively over repeated cycles, indicating increasing polarization (η) and growth of interfacial resistance due to SEI thickening on the Li anode and CEI accumulation on the LFP cathode. In contrast, the AGPE‐based cell exhibited negligible peak shifts, implying low polarization, stable Li^+^ transport, and suppressed interfacial reactions throughout cycling.^[^ ^20^, ^54^ ^]^

EIS was performed to quantitatively assess the interfacial resistance. Nyquist plots obtained after formation (Figure 6c) and after 100 cycles (Figure 6d) displayed single semicircles, corresponding to the combined resistance of the SEI film and charge transfer process (R_SEI_ + R_ct_). As R_SEI_ was on the order of a few ohms, an equivalent circuit model comprising R_b_ – (R_SEI_ + R_ct_ || CPE) – W (Warburg) was used for fitting.^[^ ^55^, ^56^ ^]^ As shown in the bar chart in Figure S26 (Supporting Information), the LE cell exhibited a substantial increase in R_SEI_ + R_ct_ after cycling, attributed to the thickening of an organic‐rich SEI layer and uneven Li deposition caused by continuous electrolyte degradation. In contrast, the AGPE cell maintained a stable hybrid SEI and uniform ion transport pathways, effectively suppressing resistance build‐up, which is consistent with the stable CV profiles.

Furthermore, SEM top‐view images (Figure 6e,f) revealed that the Li metal surface in AGPE cells remained smooth and uniform after cycling, while the LE cells displayed a rough morphology populated with dendrites and dead Li. Similarly, SEM analysis of the LFP cathode (Figure S27, Supporting Information) showed the formation of cracks between particles in LE cells after cycling, whereas the AGPE cell retained structural integrity, supporting the interpretation of lower interfacial resistance.

Figure 6g schematically illustrates the full cell configuration of the Li/AGPE/LFP battery and highlights the critical roles of the AGPE electrolyte. The continuous and rapid Li^+^ transport channels facilitated by the PDOL polymer matrix, together with the robust hybrid SEI composed of LiF, LiCl, and LiAl formed at the LMA interface, significantly enhance interfacial stability and suppress dendritic growth. These synergistic features maintain electrode integrity throughout cycling and thus enable the Li/AGPE/LFP cell to achieve exceptional long‐term cycling stability and superior high‐rate capability. This confirms the practical viability of AlCl_3_‐initiated gel polymer electrolyte in quasi‐solid state lithium metal batteries.

## 3 Conclusion

In this study, a novel polymer gel electrolyte (AGPE) based on in situ polymerized PDOL was successfully developed directly within LMB cells by employing an additive of AlCl_3_, a strong Lewis acid, as the initiator. Beyond serving as a polymerization initiator, AlCl_3_ forms multiple coordination bonds with PDOL chains, reinforcing the electrolyte framework and establishing cation‐selective Li^+^ transport pathways that promote fast, efficient Li^+^ conduction while suppressing anion mobility. As a result, the AGPE exhibited high room‐temperature ionic conductivity (≈5.0 mS cm^−1^) and an exceptionally high Li‐ion transference number (t_Li+_ = 0.75). Moreover, AlCl_3_ reacted at the Li interface to form a hybrid SEI layer composed of LiF, LiCl, and LiAl, effectively suppressing dendritic Li growth. Consequently, Li||LiFePO_4_ full cells, the AGPE demonstrated outstanding electrochemical performance, retaining 92.7% of its initial capacity after 280 cycles at 0.5 C. It also exhibits exceptional high‐rate capability, delivering a capacity of 118.2 mAh g^−1^ at a high current density of 10 C, outperforming other comparable systems.

This work demonstrates that the strong Lewis acid AlCl_3_ can play dual roles by initiating in situ polymerization and acting as a multifunctional additive that tailors the electrolyte microstructure and stabilizes interfacial reactions. These findings establish AlCl_3_‐initiated AGPE as a promising platform for the development of high‐performance, safe LMBs.

## Conflict of Interest

The authors declare no conflict of interest.

## Supporting information



Supporting Information

## Data Availability

The data that support the findings of this study are available from the corresponding author upon reasonable request.
