# Supplementary material for: A Multifunctional Potent Lewis Acid for In Situ Formation of Poly‐Dioxolane Electrolytes Toward High‐Performance Quasi‐Solid State Lithium Metal Batteries
Source: Adv Sci (Weinh). 2025 Nov 30;13(9):e19181. doi: 10.1002/advs.202519181 (PMC12904033; doi:10.1002/advs.202519181)
Supplement: Supplementary file 1 — Supporting Information [file ADVS-13-e19181-s001.docx]

Supporting Information

A Multifunctional Potent Lewis Acid for In situ Formation of Poly-Dioxolane Electrolytes toward High-Performance Quasi-Solid State Lithium Metal Batteries

Jaehyeong Yu^†^, Seochan Hong^†^, Minseon Park, Minguk Kwak, Subin Kim, Jaehyun Heo and Won Bae Kim*

J. Yu, S. Hong, M. Kwak, Prof. W. B. Kim

Department of Battery Engineering, Graduate Institute of Ferrous & Eco Materials Technology (GIFT), Pohang University of Science and Technology (POSTECH), 77 Cheongam-ro, Nam-gu, Pohang-si, Gyeongbuk-do 37673, Republic of Korea

M. Park, S. Kim, J. Heo, Prof. W. B. Kim

Department of Chemical Engineering, Pohang University of Science and Technology (POSTECH), 77 Cheongam-ro, Nam-gu, Pohang-si, Gyeongsangbuk-do 37673, Republic of Korea

**Experimental Section**

*Preparation of AGPE*

The gel polymer electrolyte (AGPE), based on poly(1,3-dioxolane) (PDOL), was synthesized via an in-situ polymerization method using a precursor mixture comprising 1,3-dioxolane (DOL, 99.5%, Thermo Scientific), lithium bis(trifluoromethanesulfonyl)imide (LiTFSI, 99%, TCI), and aluminum chloride (AlCl_3_, 99%, Sigma-Aldrich) without further purification. A 2 M LiTFSI solution in DOL was formulated through the dissolution of 1.105 g of LiTFSI into 2.0 g of DOL. Separately, a 10 mM AlCl_3_ stock solution was obtained by dissolving 2.56 mg of AlCl_3_ into 2.0 g of DOL. These solutions were subsequently mixed in predetermined proportions to adjust the final AlCl_3_ content in the precursor. The resultant solution was stirred for 30 minutes to ensure homogeneity and clarity. All synthesis procedures were conducted in a glove box filled with argon gas (H_2_O, O_2_ < 0.5 ppm).

*Material Characterization*

FTIR spectra were acquired using a PerkinElmer Spectrum Two spectrometer, while Raman measurements were carried out on a JASCO NRS-5100 system to analyze molecular structural changes from DOL to PDOL. Thermogravimetric analysis (TGA) was performed under air using a SDT Q600 (TA Instruments), with a temperature ramp of 10 °C/min up to 600 °C. Crystalline structure was identified via X-ray diffraction (XRD) using a Bruker D2 Phaser (2nd generation). Nuclear magnetic resonance (^1^H NMR, ^7^Li NMR) spectra were collected using a Bruker Avance III HD 600 instrument with DMSO-d_6_ as the solvent. MALDI-TOF MS measurements were performed using an autoflex maX (Bruker), and the obtained spectra were deconvoluted using the GUI UniDec software to determine the molecular weight distributions. Surface morphology was characterized using a field-emission scanning electron microscope (FE-SEM, Hitachi S-4800) operated at 15 kV. High-resolution transmission electron microscopy (HR-TEM) with EELS mapping (JEOL JEM-2200FS, 200 kV) was employed to investigate nanoscale morphology and elemental distribution. Additionally, atomic force microscopy (AFM, Park Systems NX-10) in Pinpoint nanomechanical mode was used to evaluate the mechanical surface properties of cycled lithium anodes inside the glove box. X-ray photoelectron spectroscopy (XPS) measurements were performed on the 10A2 beamline at PLS-II under an ultra-high-vacuum environment of roughly 1 × 10^-9^ Torr. The binding-energy scale was aligned by referencing the Au 4f transition of a gold-foil standard.

*Cathode Fabrication and Cell Assembly*

LiFePO_4_ (LFP) cathodes were prepared by mixing LFP, Super P, and polyvinylidene fluoride (PVDF) in a weight ratio of 8:1:1 with N-methyl-2-pyrrolidone (NMP) as the solvent to form a slurry. The mixture was homogenized using ball milling and subsequently cast onto carbon-coated Al foil, followed by vacuum drying. The LFP areal mass loading was approximately 5.4 mg cm^-2^. Lithium metal foil served as the counter electrode, and Whatman GF/F glass fiber was used as the separator in the assembly of coin-type 2032 cells. For liquid electrolyte (LE) cells, 150 μL of 2 M LiTFSI/DOL solution was used. In the case of AGPE cells, 150 μL of freshly mixed precursor solution was injected into the separator for in-situ polymerization. All assembly processes were performed in an argon atmosphere (H_2_O, O_2_ < 0.5 ppm), and subsequent electrochemical testing was carried out at room temperature.

*Electrochemical Measurements*

Electrochemical impedance spectroscopy (EIS) was conducted using a ZIVE LAB potentiostat over the 10 MHz to 100 kHz frequency range, applying a perturbation amplitude of 10 mV. Ionic conductivity was assessed using stainless steel symmetric cells over a temperature range of 25 ℃ to 80 ℃, calculated via the equation (1):^[1]^

$\sigma=\frac{D}{R\cdot S}$ (1)

, where D is electrolyte thickness, R the resistance, and S the electrode area.

Ion conductivity was plotted based on the following Arrhenius equation (2):^[2]^

$\sigma=Aexp(-\frac{E_{a}}{RT})$ (2)

The lithium-ion transference number (t_Li+_) was derived through potentiostatic polarization in Li || Li symmetric cells using the Vincent-Evans equation (3):^[3]^

$t_{{Li}^{+}}=\frac{I_{SS}(\Delta V-I_{0}R_{0})}{I_{0}(\Delta V-I_{SS}R_{SS})}$ (3)

ΔV, the applied polarization voltage (10 mV), was used to determine the current (I) and resistance (R) values, where I_0_ and R_0_ correspond to the initial state, and I_S_ and R_S_ indicate the steady-state after polarization. Linear sweep voltammetry (LSV) was recorded from 3.0 to 5.5 V at 1 mV s^-1^. Tafel plots were obtained within ±0.1 V vs. Li/Li^+^ at 1 mV s^-1^ to evaluate charge transfer kinetics. Cyclic voltammetry (CV) was conducted at 0.2 mV s^-1^ over 2.5-4.0 V, and galvanostatic cycling of Li || LFP cells was performed between 2.5 and 3.8 V at 0.5 C using a WBCS 3000 system (WonATech).

*Computational methods.*

Electronic and structural calculations were carried out via density functional theory (DFT). Binding energy and frontier molecular orbital (HOMO–LUMO) analysis were performed using the General Atomic and Molecular Electronic Structure System (GAMESS) with the Becke three-parameter hybrid functional combined with the Lee–Yang–Parr correlation functional (B3LYP) and a 6-31G** basis set.^[4,5]^ Geometry optimizations utilized the Restricted Hartree–Fock (RHF) method with a convergence criterion of 0.0001 Hartree/Bohr and up to 500 steps. Huckel guess was used to initiate orbital generation.^[6]^ Binding energies (BE) were computed using the following equation (4):

BE=E_A_​+E_B_​−E_AB_ (4)

Positive BE values indicate favorable interactions.^[7]^ Molecular orbitals were visualized using VESTA. Adsorption and diffusion energy barrier calculations were conducted using the Quantum ESPRESSO suite with the Perdew–Burke–Ernzerhof (PBE) exchange–correlation functional within the generalized gradient approximation (GGA), applying Projector Augmented Wave (PAW) pseudopotentials.^[8]^ The structure relaxation was performed using the Broyden–Fletcher–Goldfarb–Shanno (BFGS) algorithm, with energy and force convergence thresholds of 1.0 × 10^-7^ Ry and 0.00038 Ry/Bohr, respectively.^[9]^ The energy cutoffs were 30 Ry for wavefunctions and 180 Ry for charge density, with a 3×3×1 Monkhorst-Pack k-point mesh and DFT-D3 (BJ damping) for van der Waals corrections.^[10]^ The slab models were constructed using unit cell data from the Materials Project database, incorporating a >10 Å vacuum layer along the z-axis.^[11]^ The bottom slab layers were held fixed, and the top layers allowed to fully relax. Adsorption energy (AE) was determined by following equation (5):

AE=E_AB_​−E_A_​−E_B_​ (5)

We calculated the diffusion barriers using the nudged elastic band (NEB) approach.^[12]^ All structure visualizations were rendered via VESTA. Simulations were executed through the cloud-based platform Materials Square.


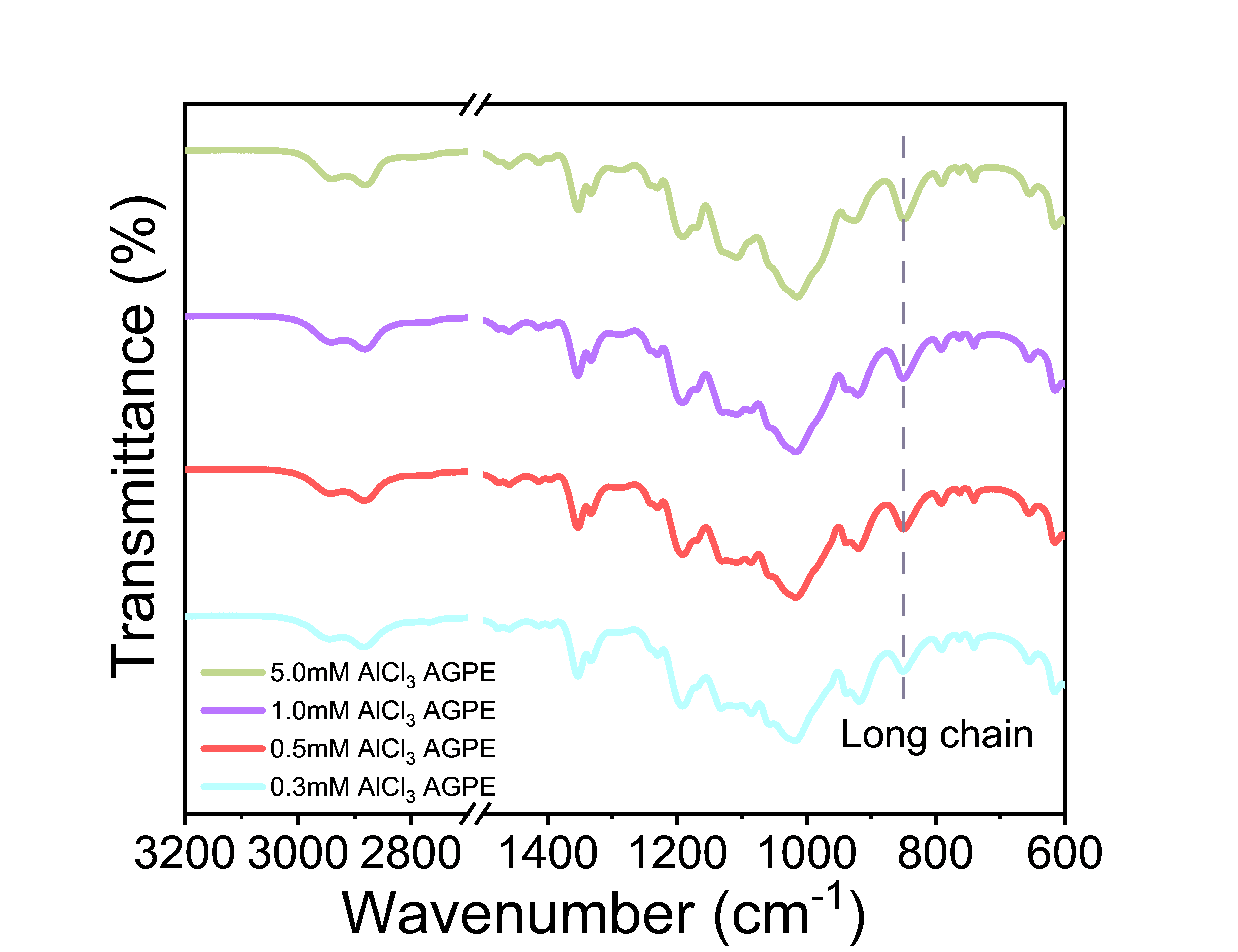


**Figure S1**. FTIR spectra of AGPE formed at different AlCl_3_ concentrations.


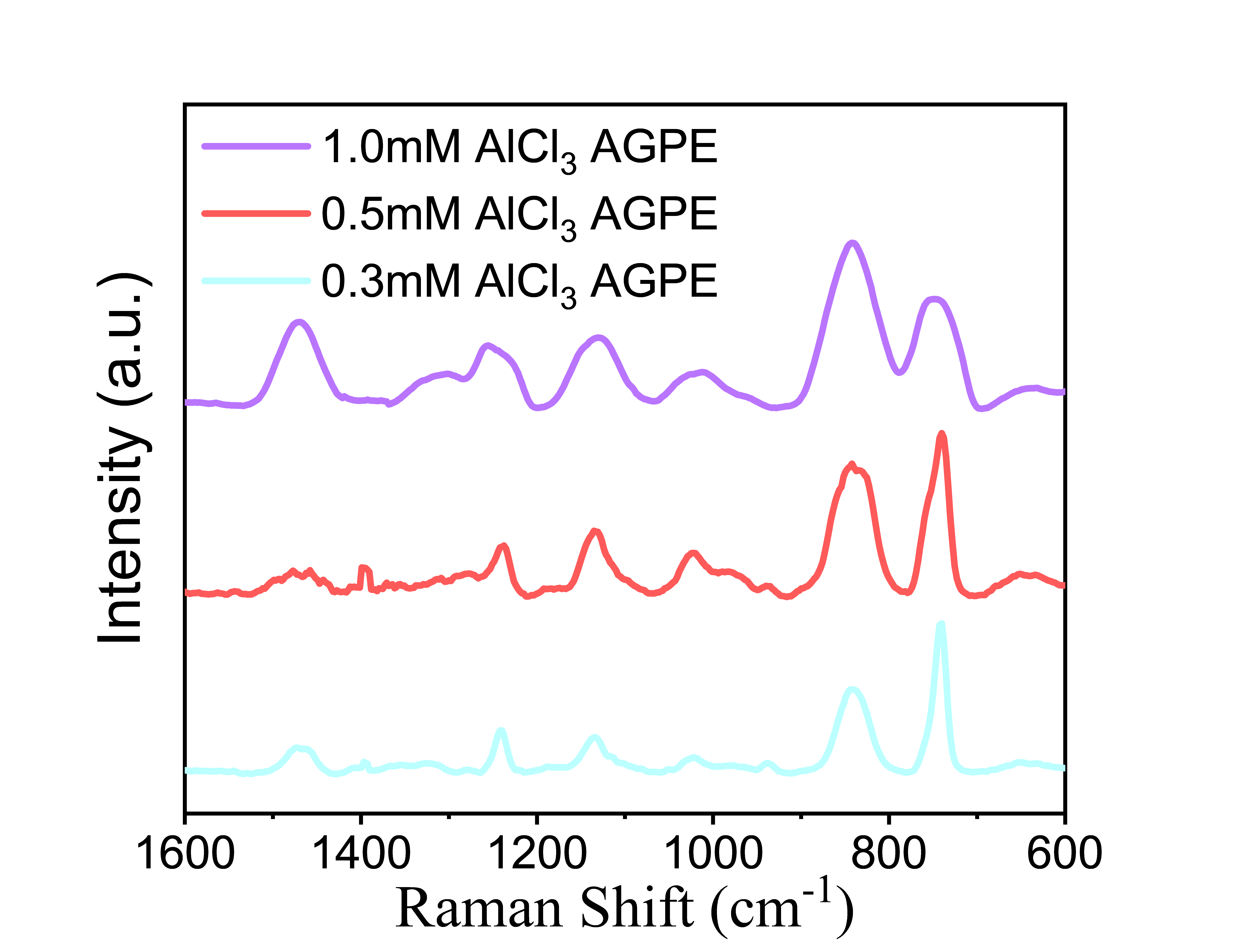


**Figure S2**. Raman spectra of AGPE formed at different AlCl_3_ concentrations.


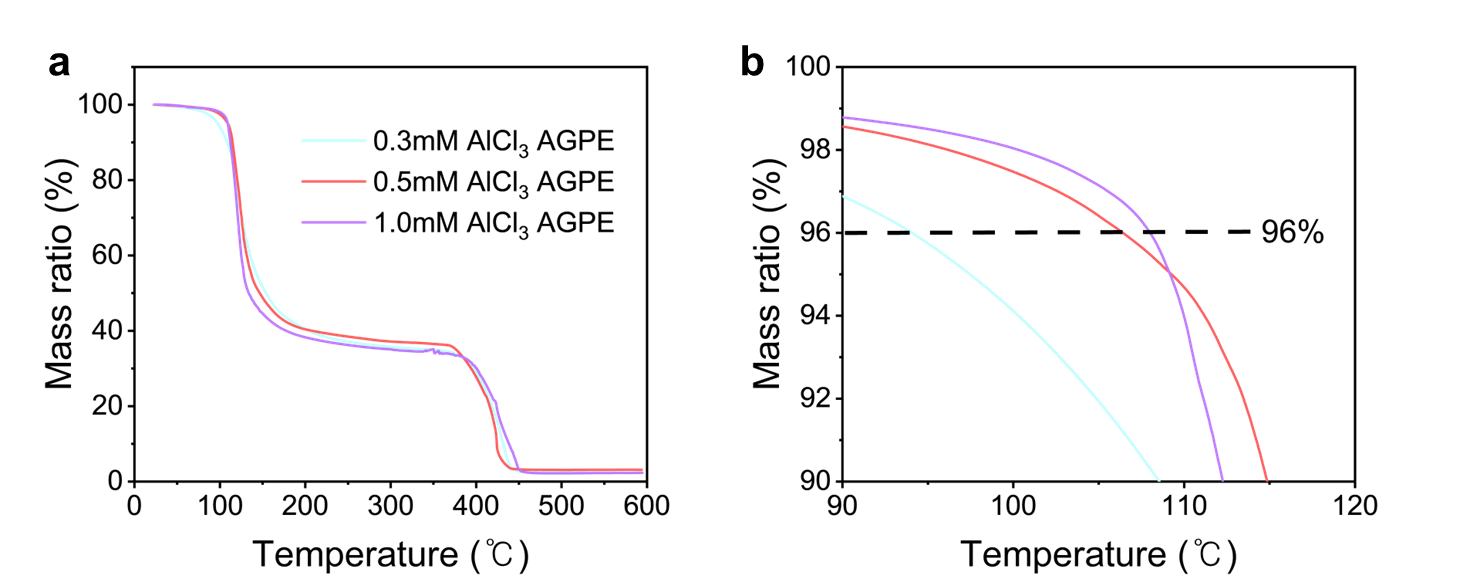


**Figure S3**. (a) TGA curves of AGPE formed at different AlCl_3_ concentrations. (b) Enlarged TGA curves of AGPE around 96% mass retention region.


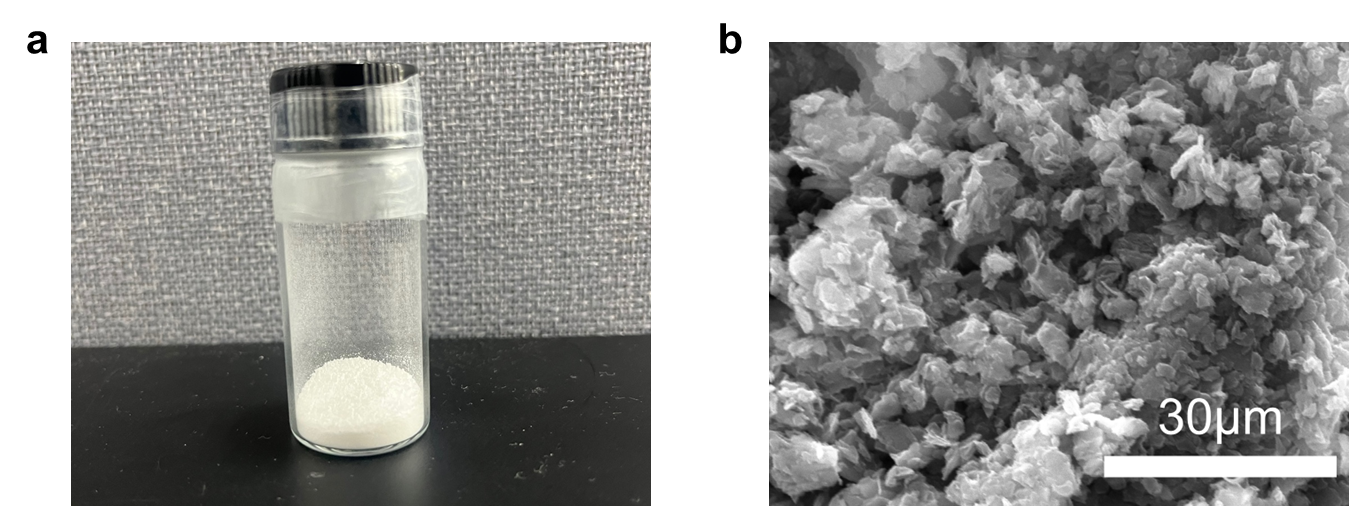


**Figure S4**. (a) Optic image of PDOL powder after washing with D.I. water. (b) SEM image of PDOL powder.

**
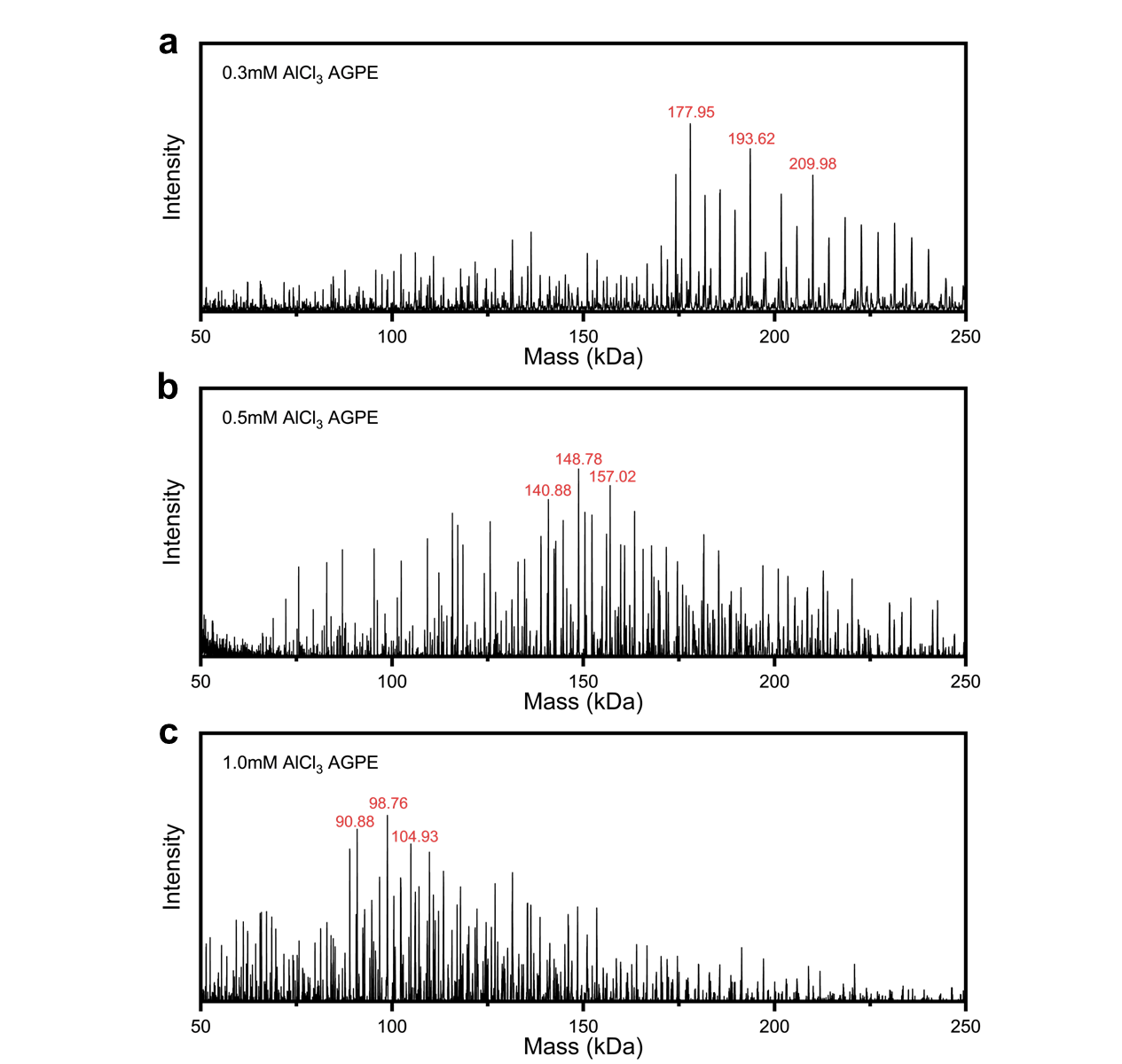
**

**Figure S5**. MALDI-TOF spectra of AGPE with different initiator concentrations.


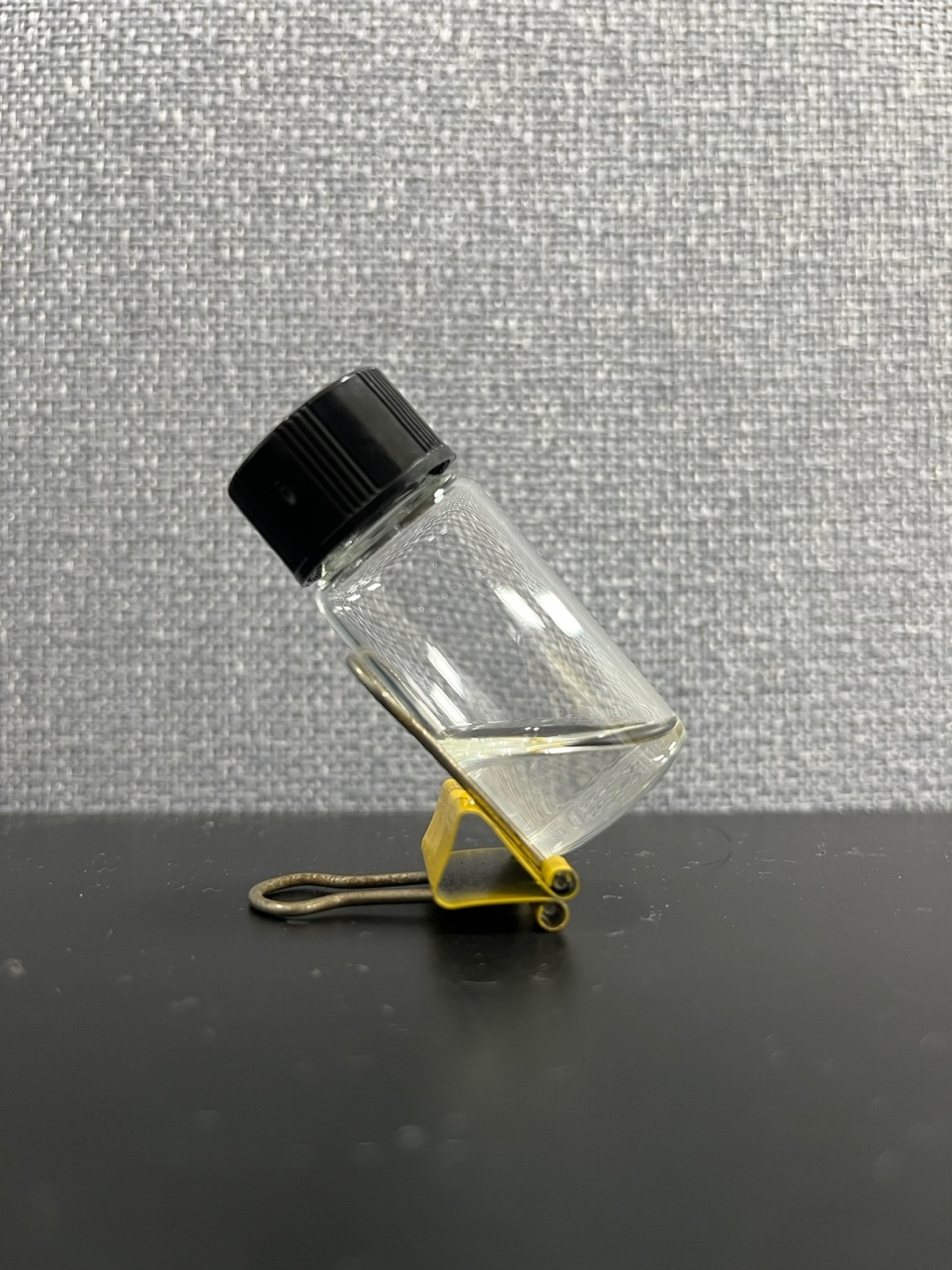


**Figure S6**. Optic image of 10mM AlCl_3_ / DOL without LiTFSI after 24h at room temperature.


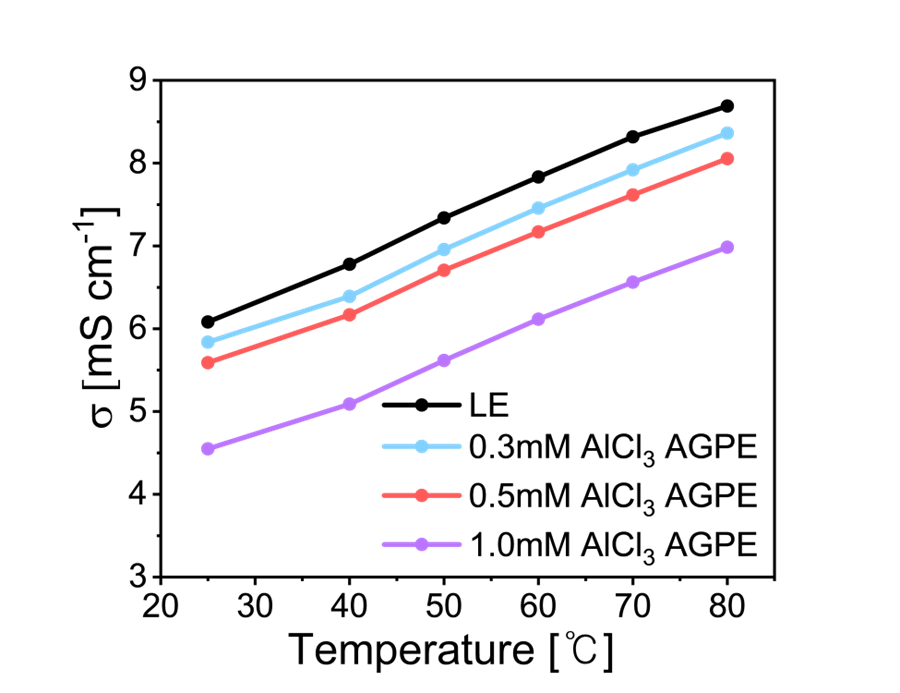


**Figure S7**. Ion conductivity [mS cm^-1^] via temperature [℃] of LE and AGPE formed at different AlCl_3_ concentrations.


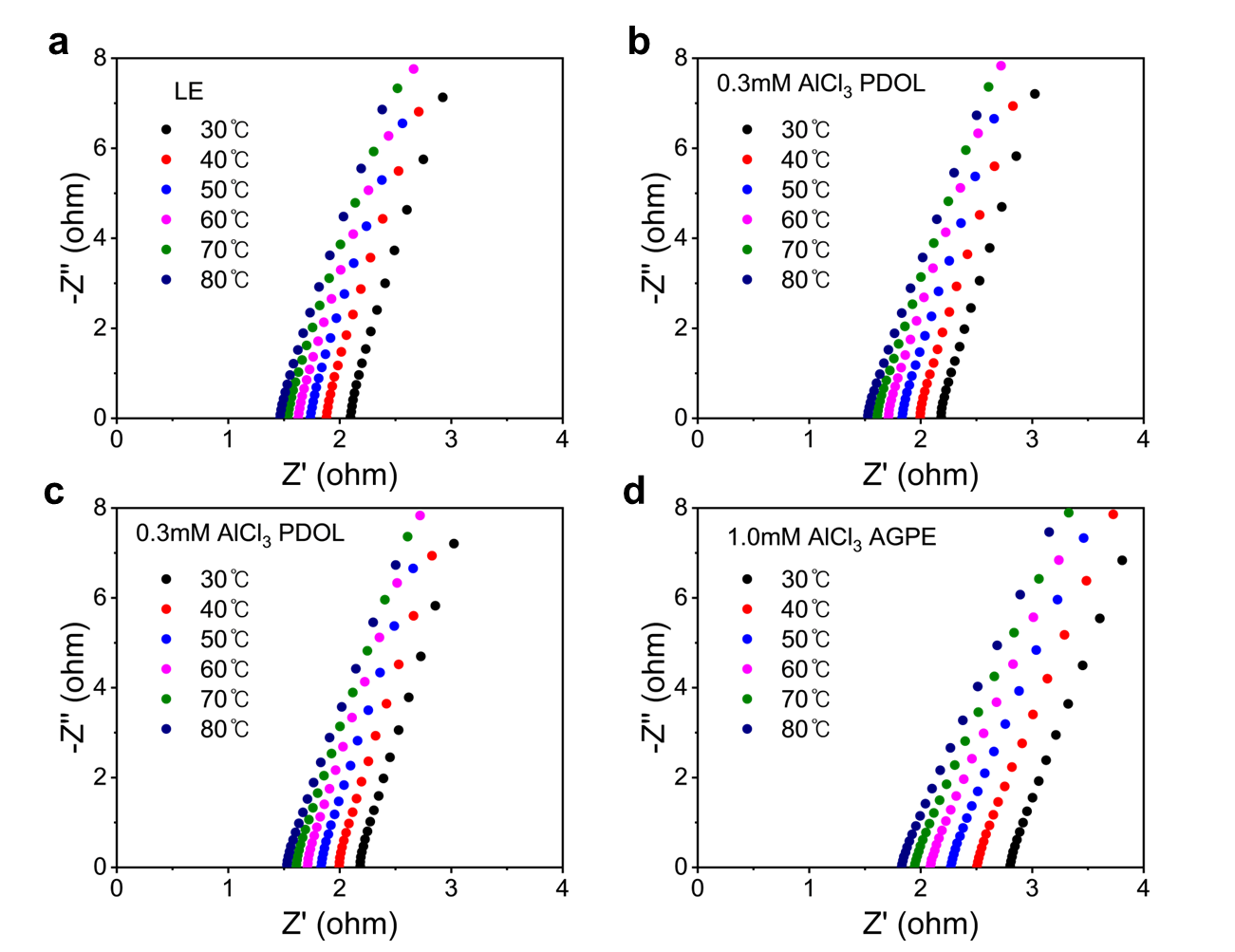


**Figure S8**. EIS spectra of (a) LE, (b) 0.3 mM AlCl_3_ AGPE, (c) 0.5 mM AlCl_3_ AGPE and (d) 1.0 mM AlCl_3_ AGPE at various temperatures.


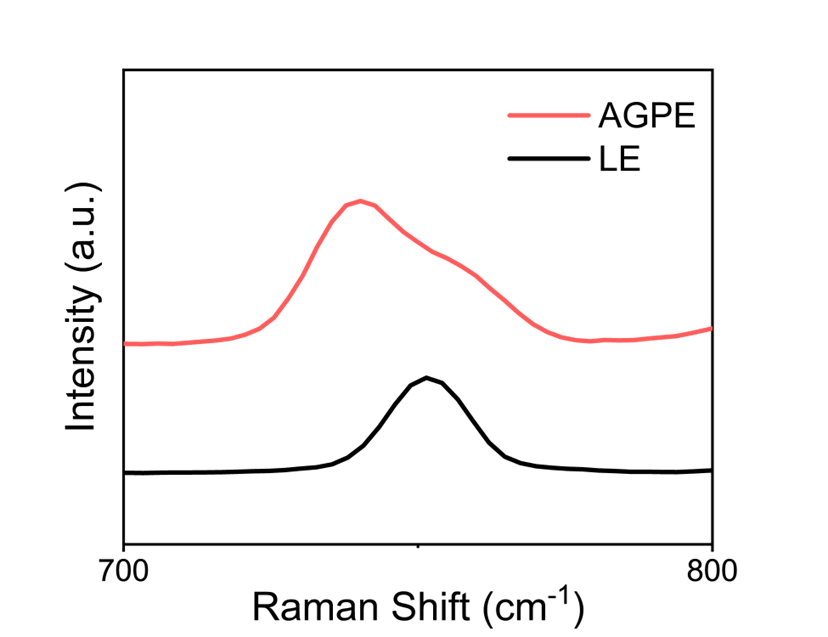


**Figure S9**. Raman spectra of LE and AGPE at low Raman shift area between 700 cm^-1^ and 800 cm^-1^.


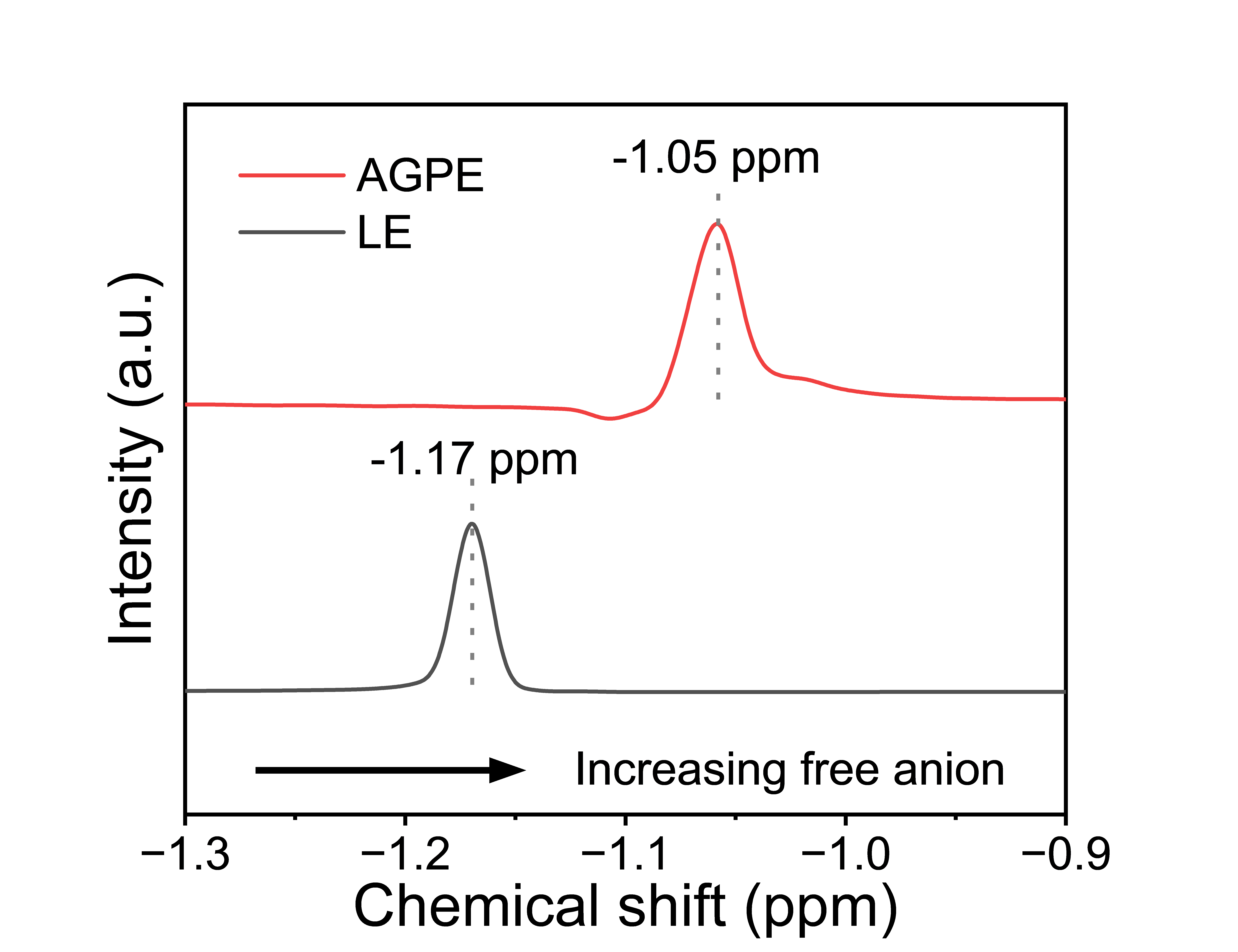


**Figure S10**. ^7^Li NMR spectra of LE and AGPE.


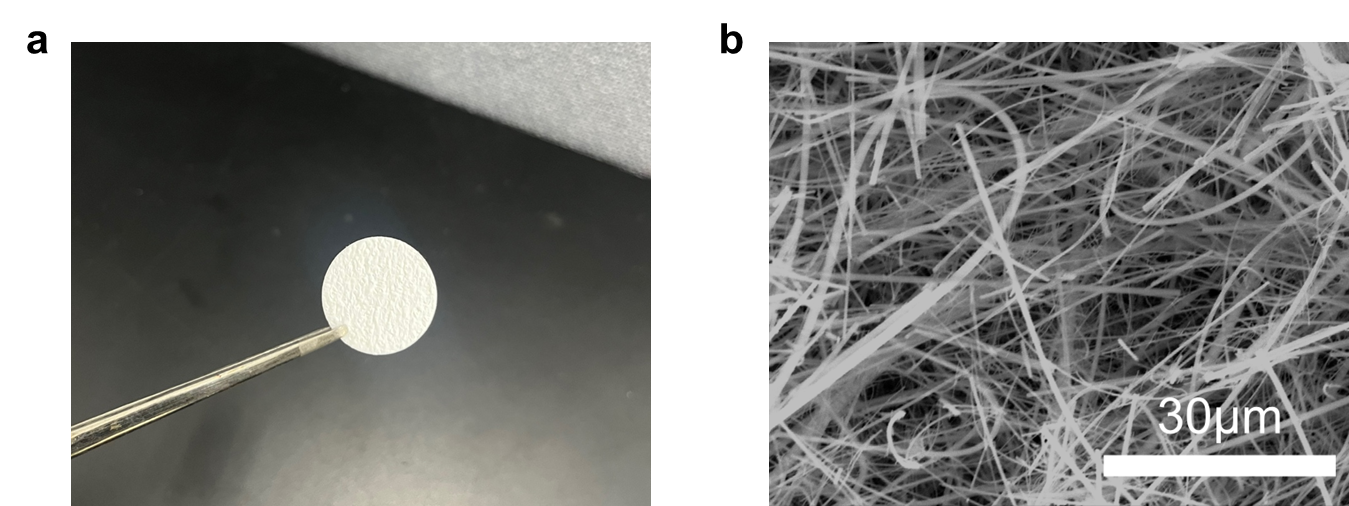


**Figure S11**. (a) Optic image of glass fiber separator. (b) SEM image of glass fiber separator.


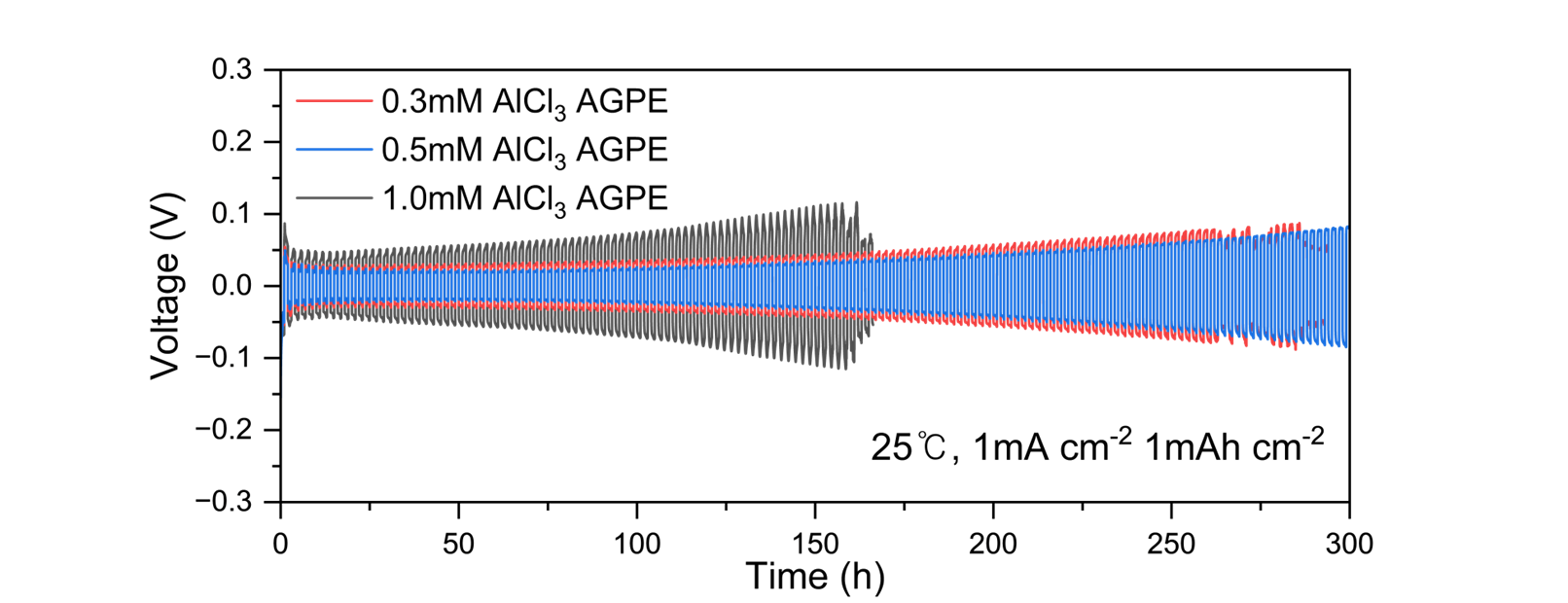


**Figure S12**. Li plating/stripping profiles of Li || Li symmetric cells assembled with AGPE formed at different AlCl_3_ concentrations under 1mA cm^-2^.


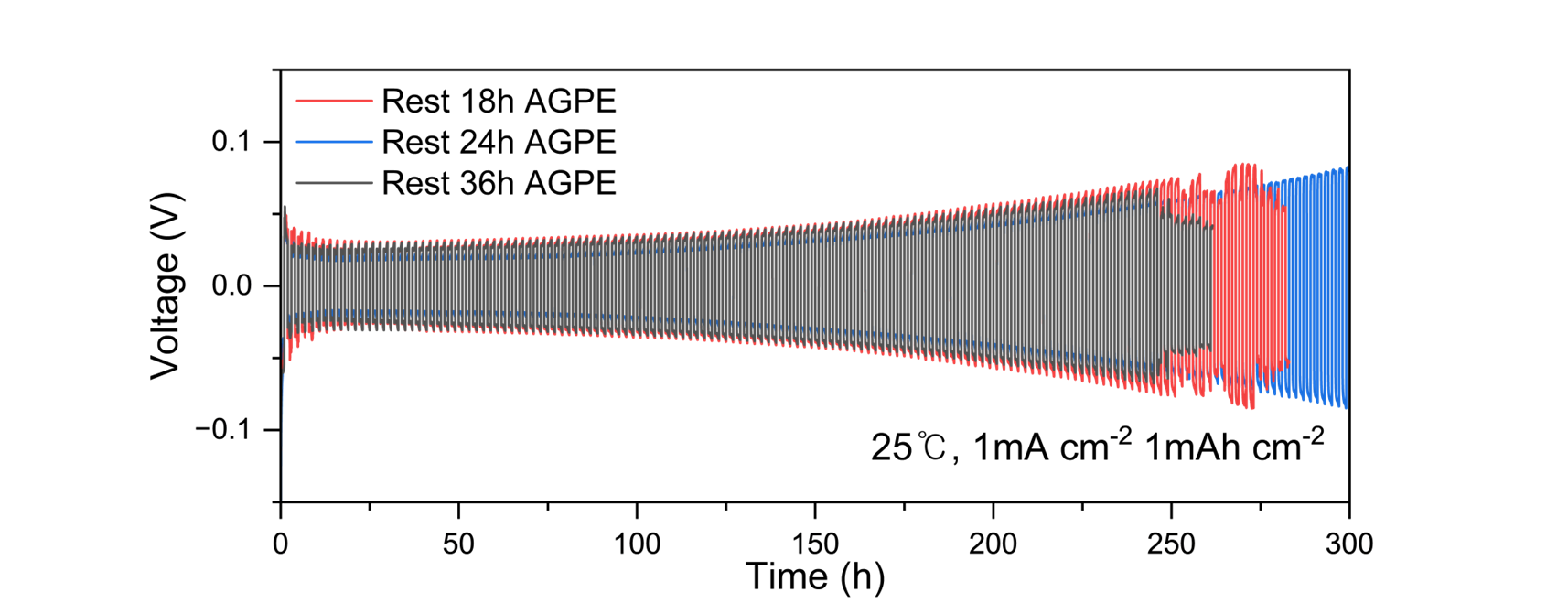


**Figure S13**. Li plating/stripping profiles of Li || Li symmetric cells assembled with AGPE formed after different rest time under 1mA cm^-2^.


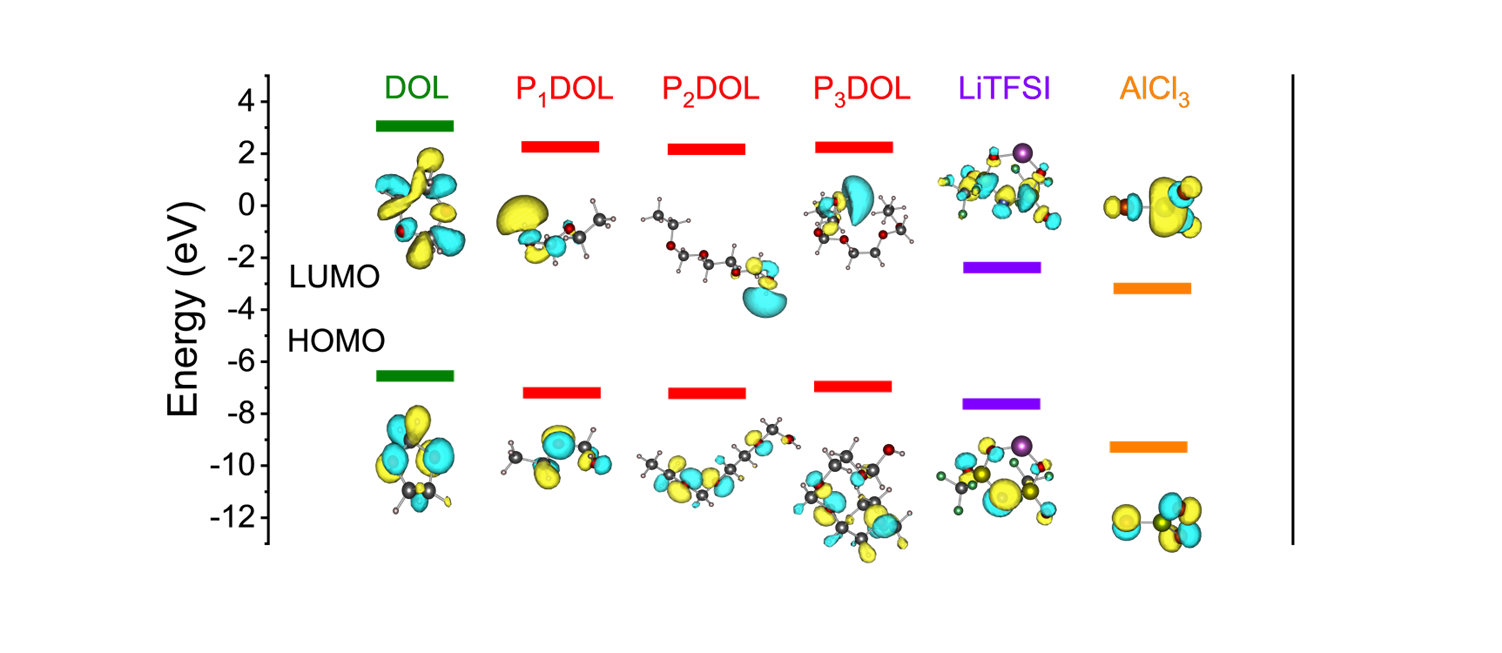
**Figure S14**. Calculated highest occupied molecular orbital (HOMO) and lowest unoccupied molecular orbital (LUMO) energy levels of DOL, P_1_DOL, P_2_DOL, P_3_DOL, LiTFSI, AlCl_3_.


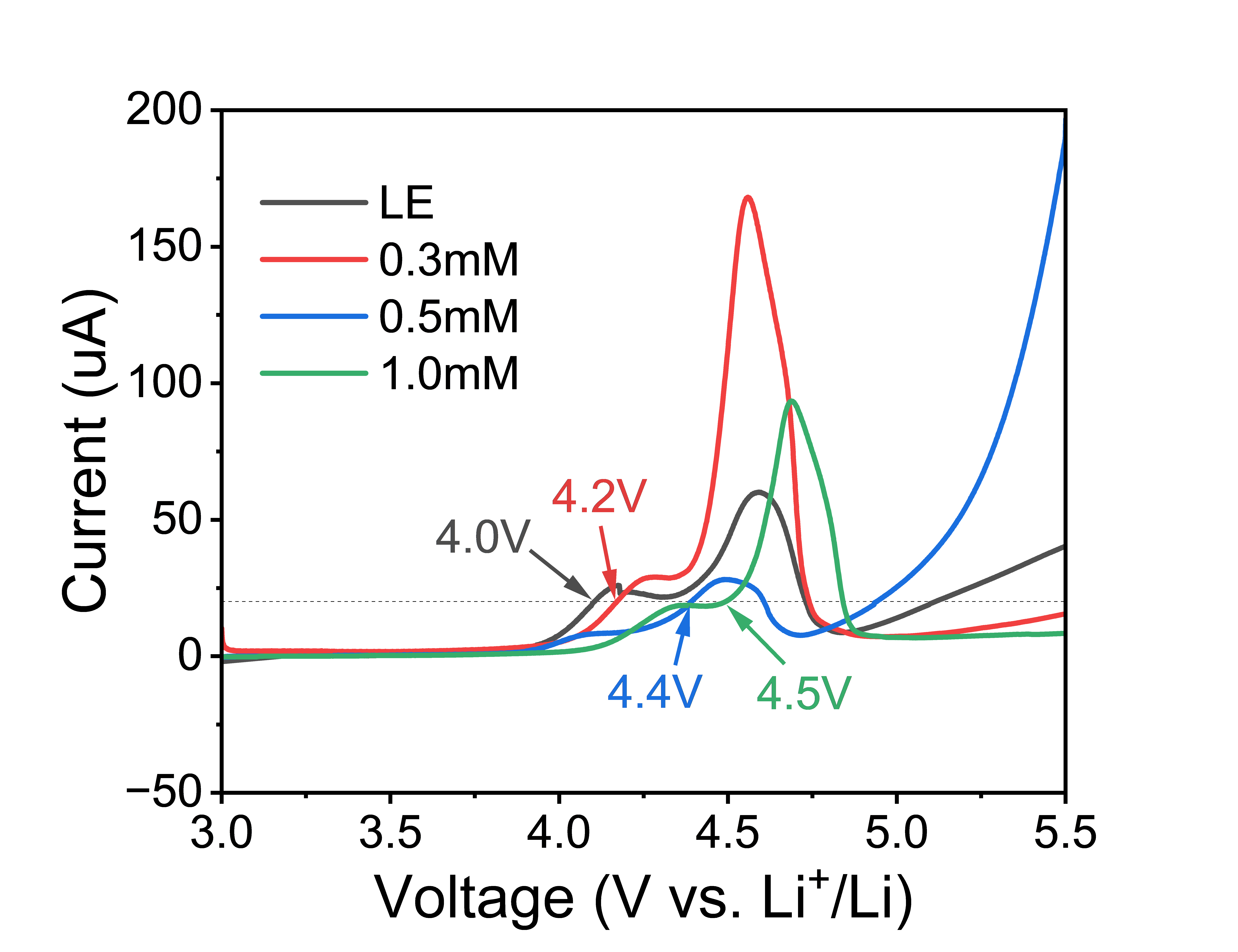


**Figure S15**. Linear sweep voltammograms of AGPE at different AlCl_3_ concentrations.

**
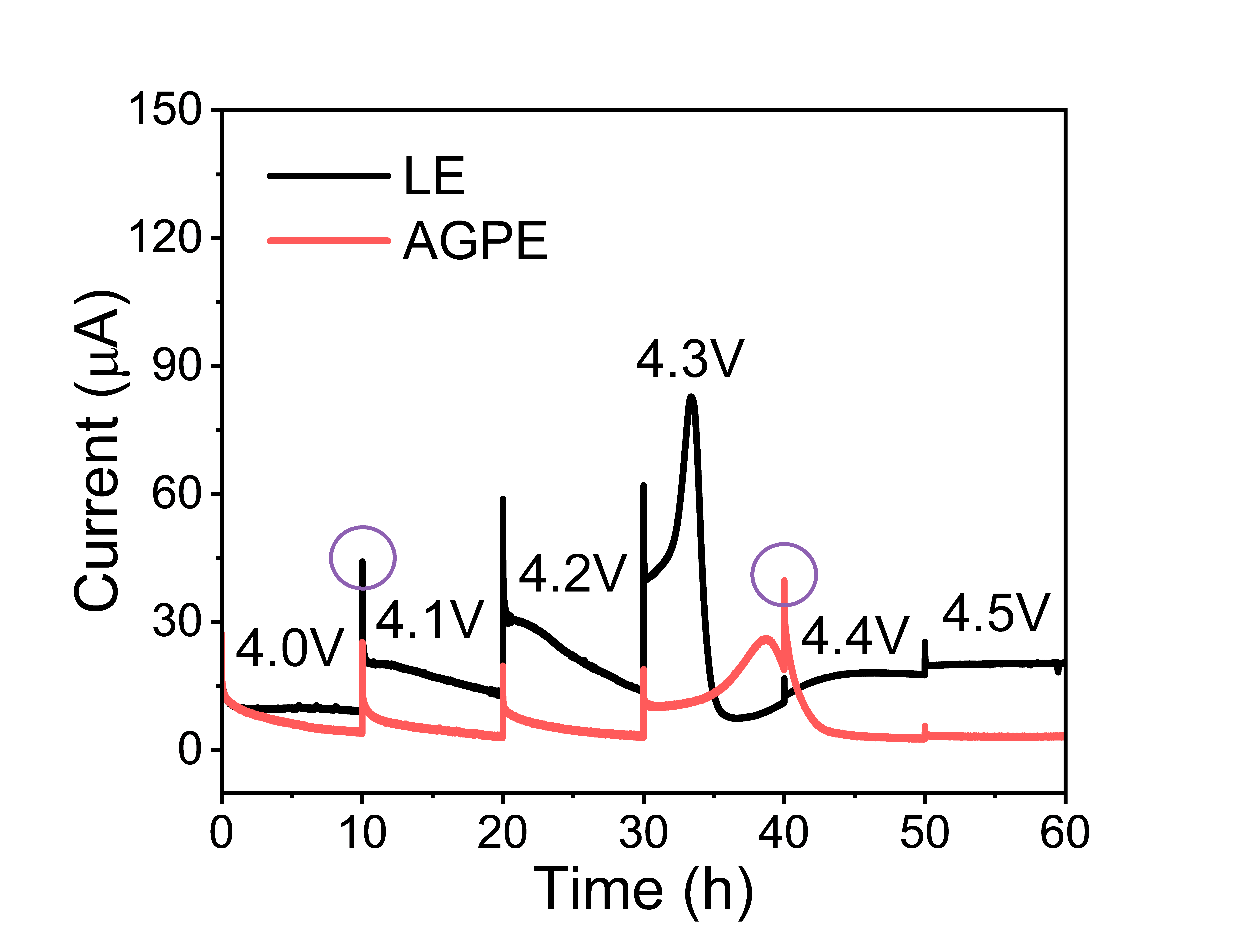
**

**Figure S16**. Electrochemical floating analysis of cells using Li/SUS cell for LE and AGPE.


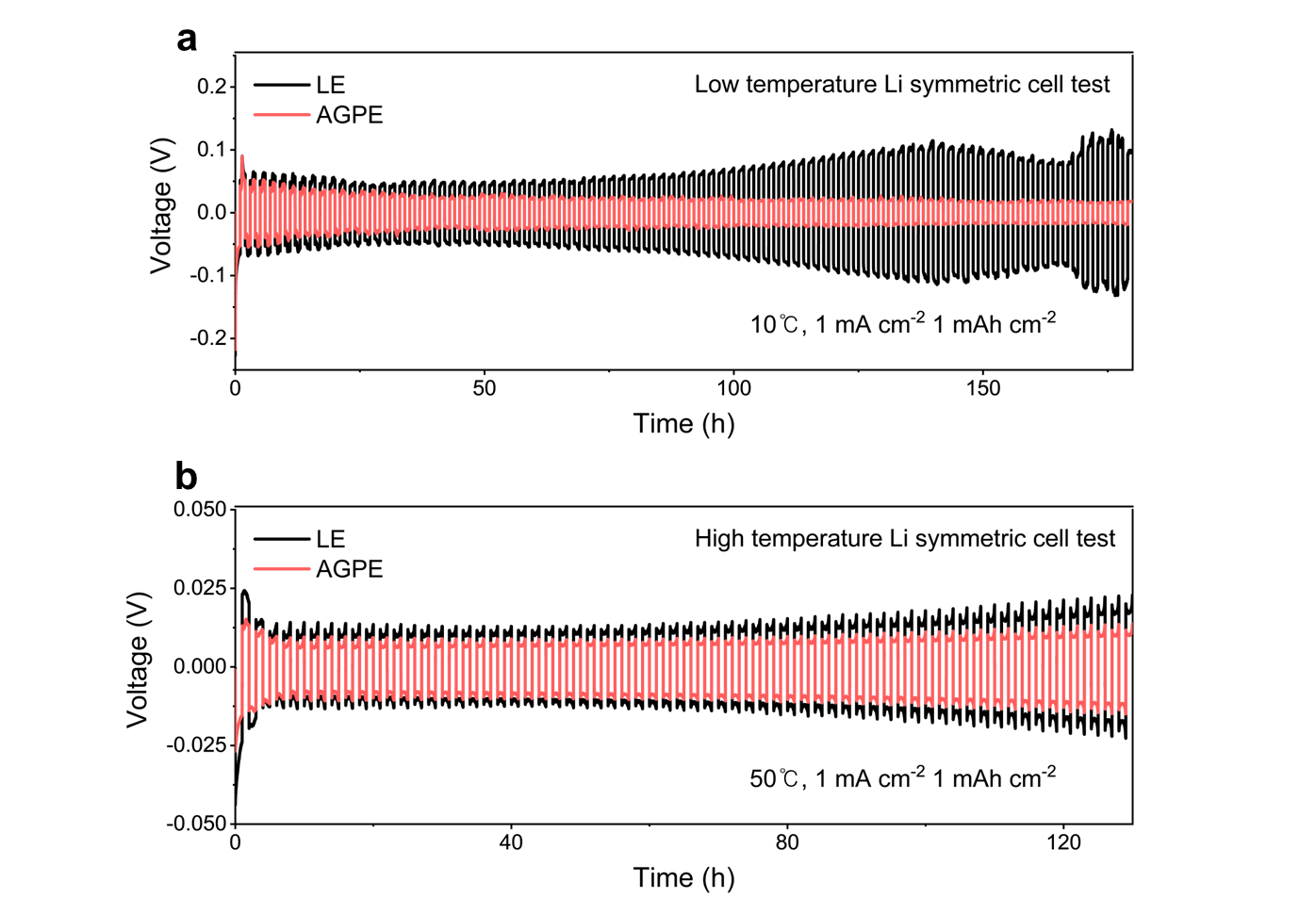


**Figure S17**. Li plating/stripping profiles of Li || Li symmetric cells assembled with LE and AGPE under 1 mA cm^-2^ at (a) 10 °C and (b) 50 °C


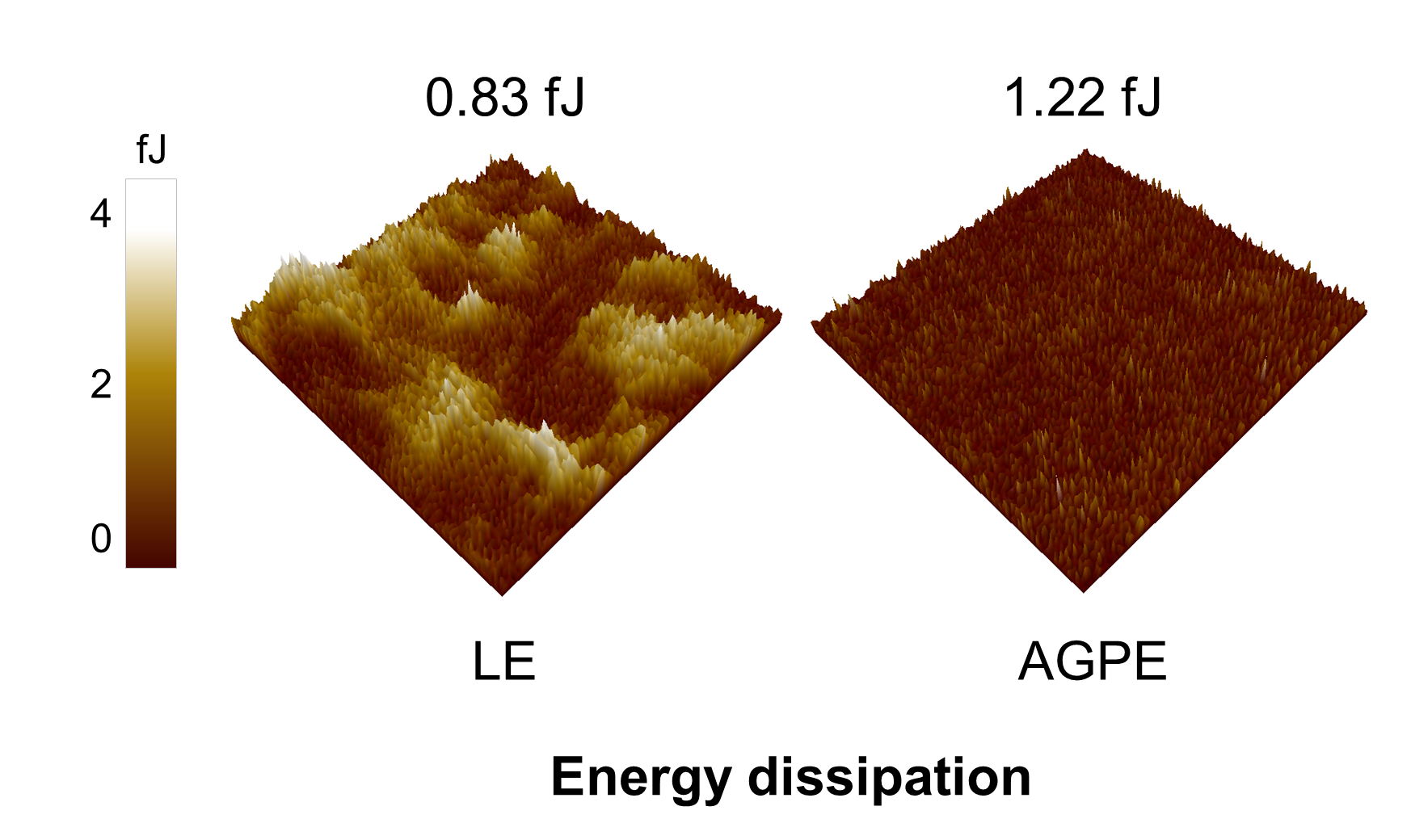


**Figure S18**. AFM characterization of the energy dissipation mapping of Li metal anode after cycling Li || Li symmetric cells assembled by LE and AGPE.


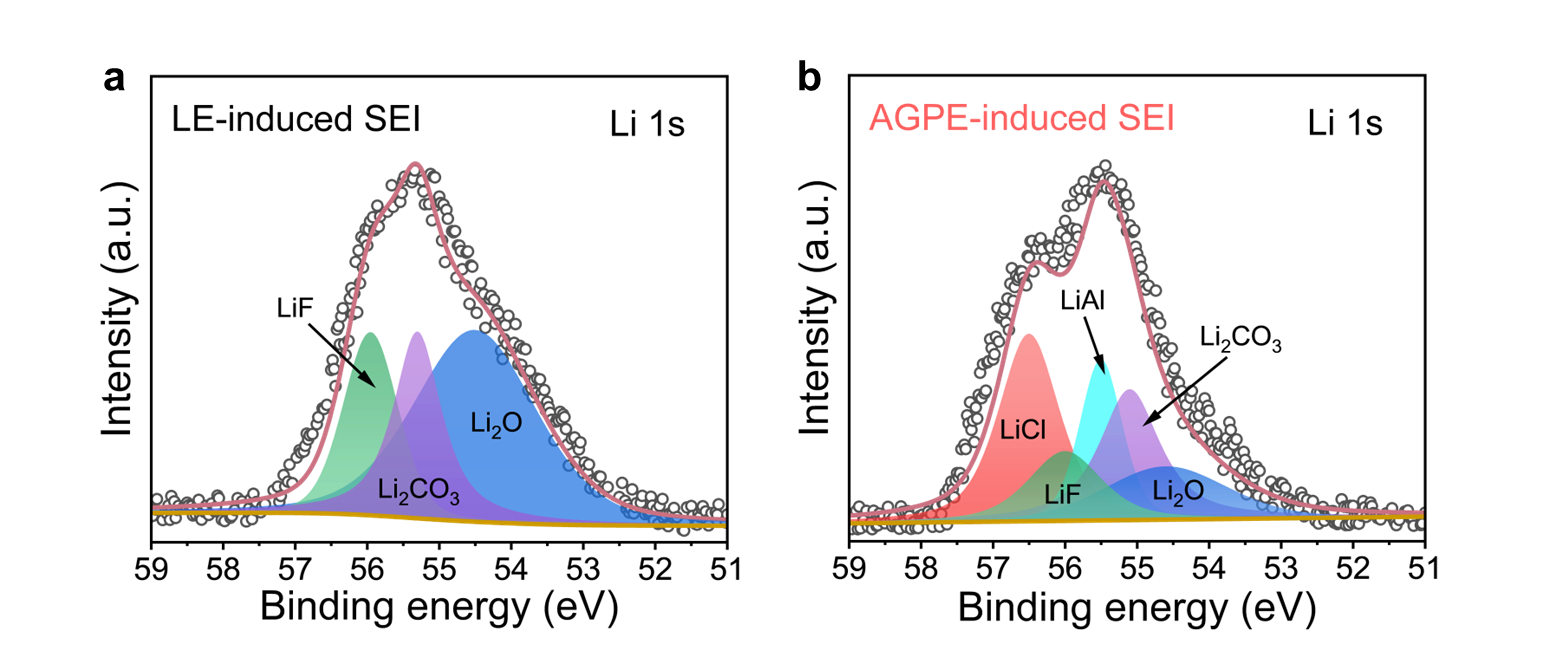


**Figure S19**. XPS spectra of Li 1s taken from cycled Li metal surface with (a) LE and (b) AGPE after 100 cycles.


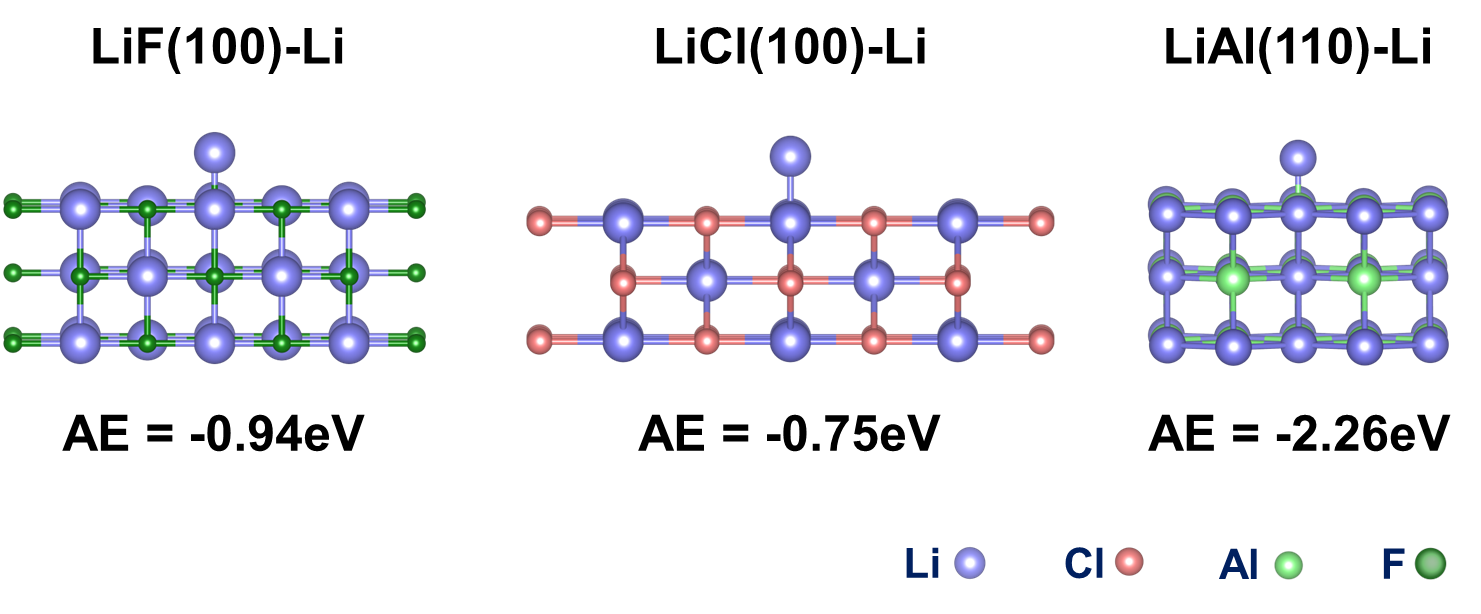


**Figure S20**. The schematic images of adsorption energy of Li^+^ on the LiF (100), LiCl (100), and LiAl (110) surfaces.


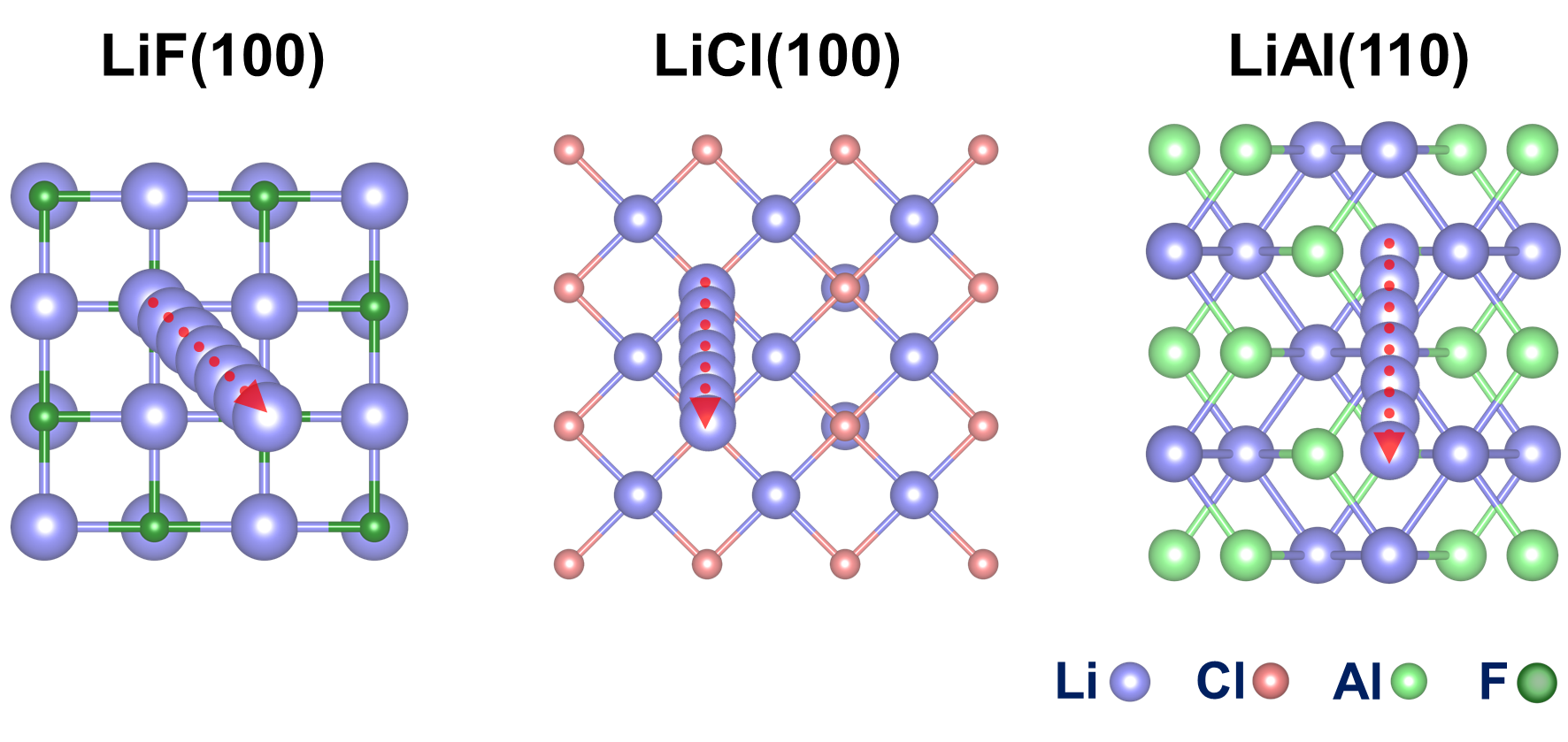


**Figure S21**. The schematic images of Li^+^ diffusion path on the LiF (100), LiCl (100), and LiAl (110).


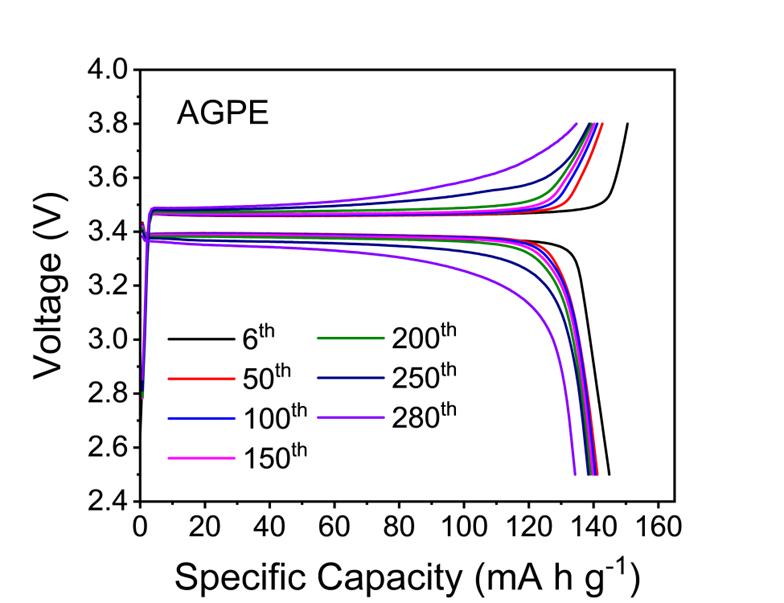


**Figure S22**. Charge/discharge curves of Li/AGPE/LFP cells at diﬀerent cycles.

**

**

**Figure S23**. Cyclic performance of Li/LE/LFP cells and Li/AGPE/LFP cells at 1.0 C


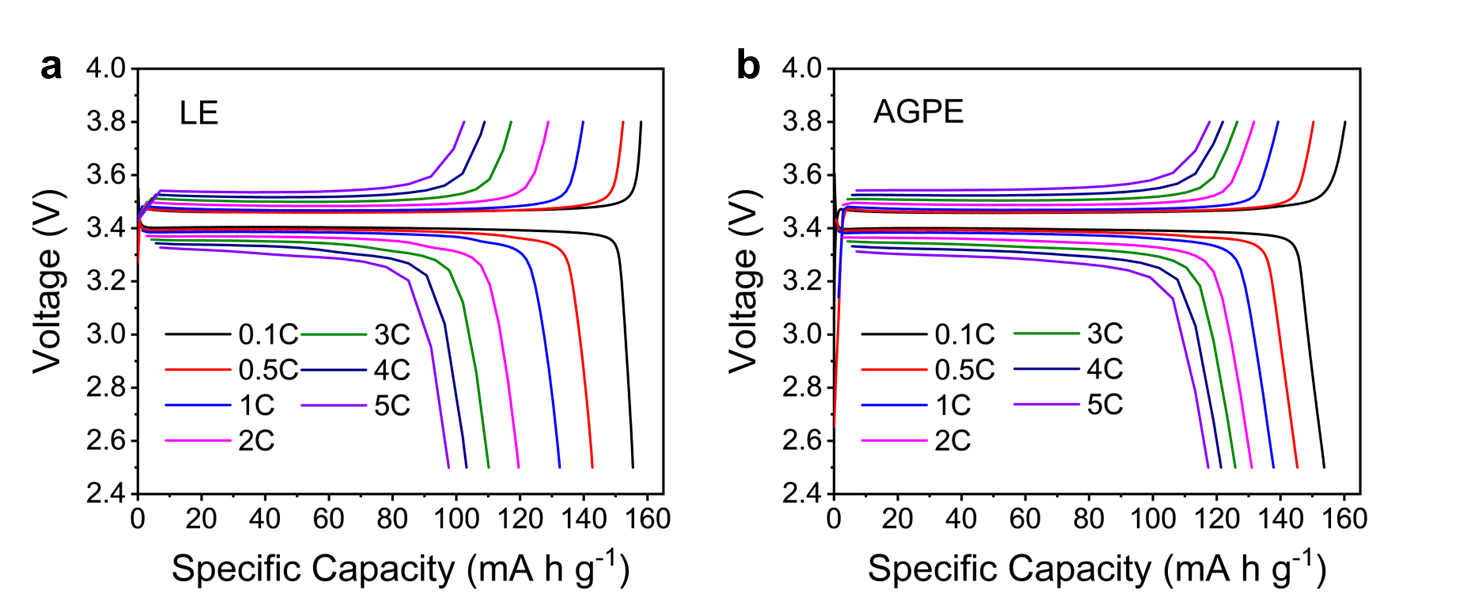


**Figure S24**. Charge/discharge curves of (a) Li/LE/LFP cells and (b) Li/AGPE/LFP cells at diﬀerent C-rates.


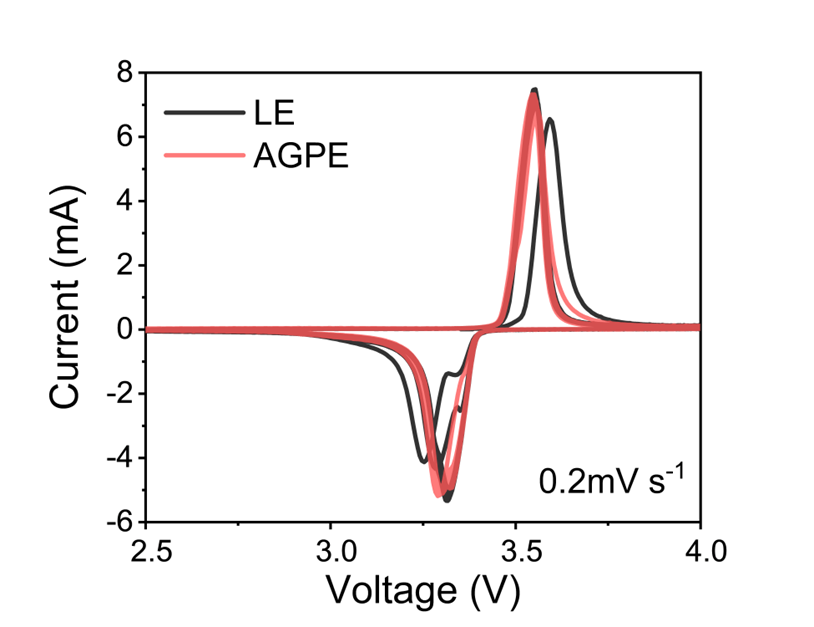


**Figure S25**. CV curves of Li/LE/LFP cells and Li/AGPE/LFP cells at 0.2 mV s^-1^.


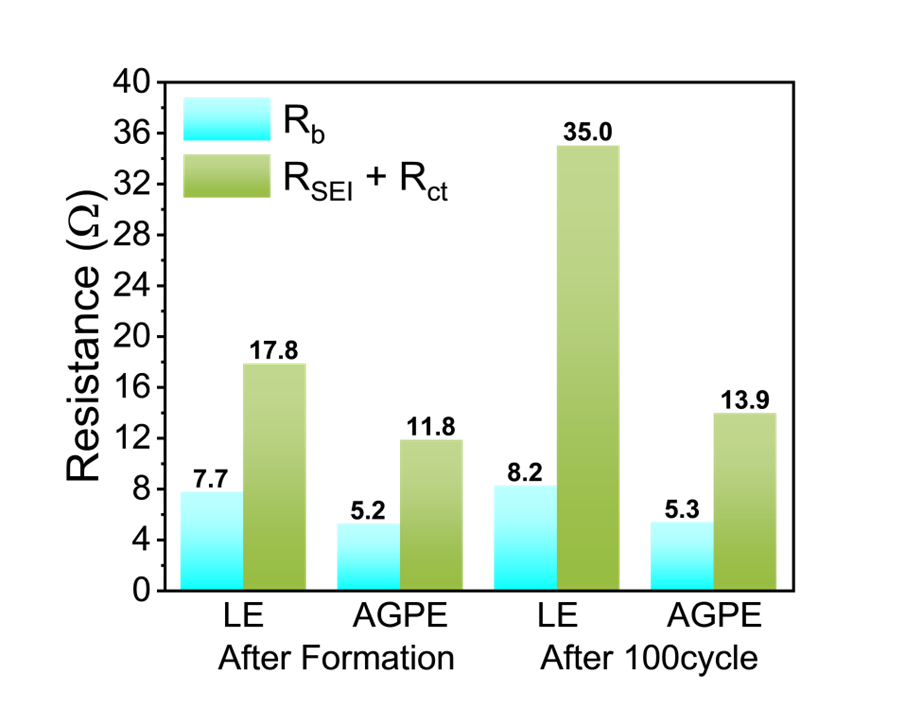


**Figure S26**. EIS-Derived Resistances of Li || LFP Cells with LE and AGPE after Formation and the 100^th^ Cycle.


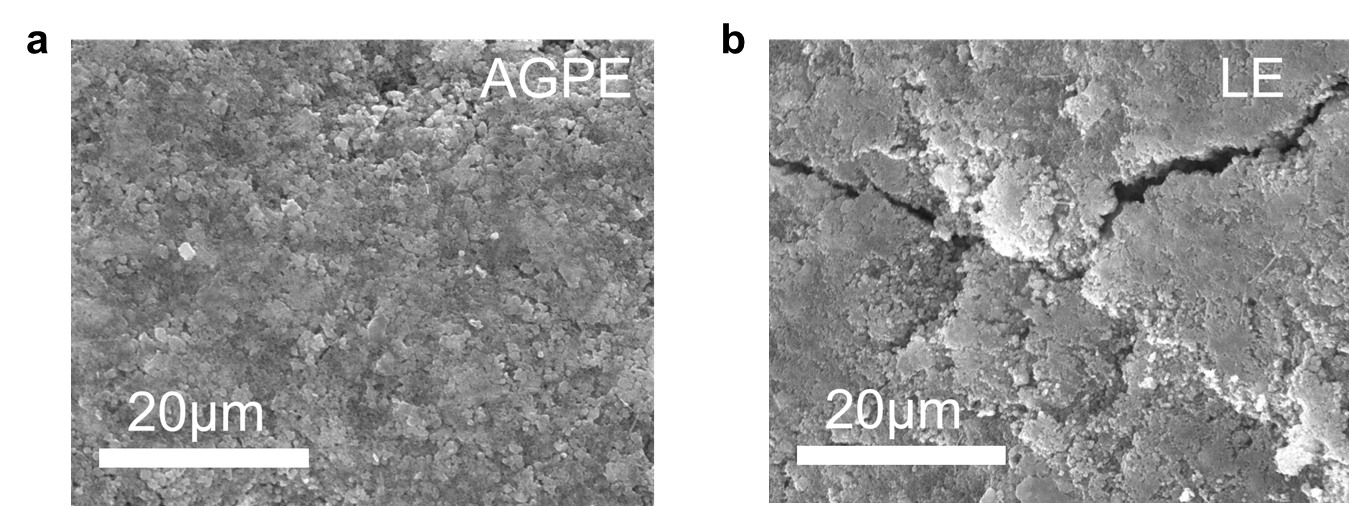


**Figure S27**. Top view SEM images of LFP cathode after cycling Li || LFP cell assembled by (a) LE and (b) AGPE.

**Figure S28**. Comparison of the Li || LFP cell rate capability performances of our work with recent PDOL based Li metal batteries. ^[4,6,13-19]^

**Table S1**. Resistance of Li || Li symmetric cells after cycling.

| **After 1 cycle** | | | | **After 100 cycles** | | | |
| --- | --- | --- | --- | --- | --- | --- | --- |
| LE | | AGPE | | LE | | AGPE | |
| Resistance | (Ω) | Resistance | (Ω) | Resistance | (Ω) | Resistance | (Ω) |
| R_b_ | 3.01 | R_b_ | 2.83 | R_b_ | 7.19 | R_b_ | 4.04 |
| R_SEI_ | 11.97 | R_SEI_ | 9.03 | R_SEI_ | 15.89 | R_SEI_ | 11.89 |
| R_ct_ | 11.85 | R_ct_ | 9.29 | R_ct_ | 15.70 | R_ct_ | 11.40 |

**Table S2**. Comparison of Adsorption energy of Li^+^ on different sites of LiF (100), LiCl (100), LiAl (110).

| **LiF (100)** | | **LiCl (100)** | | **LiAl (110)** | |
| --- | --- | --- | --- | --- | --- |
| Site | AE (eV) | Site | AE (eV) | Site | AE (eV) |
| Li top | -0.64119 | Li top | -0.62191 | Li top | -2.25714 |
| F top | -0.94322 | F top | -0.75159 | F top | -2.25708 |
| Hollow | -0.65350 | Hollow | -0.62197 | Hollow | -2.25710 |

**Table S3**. Comparison of the Li || LFP cell rate capability performances of our work with recent PDOL based Li metal batteries.

| Materials | Mass loading (mg cm^-2^) | C-rate | Rate capability (mAh g^-1^) | Ref. |  |
| --- | --- | --- | --- | --- | --- |
| AGPE | 5.4 | 5 | 118.2 | This work |  |
| PDOL@LiNO_3_ | | 3 | 2 | 125 | 4 |
| PDOL@SbF_3_ | 2.5 | 2 | 85.96 | 6 |  |
| PDOL@FTEP | | 3.2 | 5 | 90 | 13 |
| PDOL@PDA/PVDF-HFP | | 2.2 | 5 | 92 | 14 |
| PDOL@SL | 1.2 | 5 | 99.6 | 15 |  |
| PDOL@LAP | 2.5 | 4 | 99 | 16 |  |
| PDOL@Al_2_O_3_ | 5 | 3 | 95 | 17 |  |
| PDOL@PVDF-BN | 2.3 | 5 | 69.9 | 18 |  |
| PDOL@LLTO NF | 3 | 2 | 132.8 | 19 |  |

**References**

[1] J. Zhu; J. Zhang; R. Zhao; Y. Zhao; J. Liu; N. Xu; X. Wan; C. Li; Y. Ma; H. Zhang; Y. Chen. In Situ 3D Crosslinked Gel Polymer Electrolyte for Ultra-Long Cycling Lithium Metal Batteries. *Energy Storage Mater.* **2023**, 57, 92–101.

[2] C.-Z. Zhao; Q. Zhao; X. Liu; J. Zheng; S. Stalin; Q. Zhang; L. A. Archer. Rechargeable Lithium Metal Batteries with an In-Built Solid-State Polymer Electrolyte and a High Voltage/Loading Ni-Rich Layered Cathode. *Adv. Mater.* **2020**, 32, 1905629.

[3] J. Y. Liang; X. D. Zhang; Y. Zhang; L. B. Huang; M. Yan; Z. Z. Shen; R. Wen; J. Tang; F. Wang; J. L. Shi; L. J. Wan; Y. G. Guo. Cooperative Shielding of Bi-Electrodes via In Situ Amorphous Electrode–Electrolyte Interphases for Practical High-Energy Lithium-Metal Batteries. *J. Am. Chem. Soc.* **2021**, 143, 16768–16776.

[4] Q. Wang; Y. Ma; Y. Wang; X. He; D. Zhang; Z. Li; H. Sun; Q. Sun; B. Wang; L. Z. Fan. In Situ Catalytic Polymerization of LiNO_3_-Containing PDOL Electrolytes for High-Energy Quasi-Solid-State Lithium Metal Batteries. *Chem. Eng. J.* **2024**, 484, 149757.

[5] K. Mu; W. Dong; W. Xu; Z. Song; R. Wang; L. Wu; H. Li; Q. Liu; C. Zhu; J. Xu; L. Tian. In Situ Hybrid Crosslinking Polymerization of a Composite Polymer Electrolyte for Stable Lithium Metal Batteries. *Adv. Funct. Mater.* **2024**, 34, 2405969.

[6] H. Wu; Y. Lin; Z. Wang; K. Shi; J. Li; J. Pan; J. Ren; X. Li; Y. Min; M. Wu; Q. Liu. In-Situ Polymerized Electrolyte from a Bifunctional Additive for Li^+^ Bulk Phase Migration and High-Flux Interface Transport in Lithium Metal Batteries. *Chem. Eng. J.* **2024**, 486, 150343.

[7] J. Hu; C. Lai; K. Chen; Q. Wu; Y. Gu; C. Wu; C. Li. Dual Fluorination of Polymer Electrolyte and Conversion-Type Cathode for High-Capacity All-Solid-State Lithium Metal Batteries. *Nat. Commun.* **2022**, 13, 7914.

[8] J. Zhu; J. Luo; J. Li; S. Huang; H. Geng; Z. Chen; L. Jia; Y. Fu; X. Zhang; X. Zhuang. A Porous Li–Al Alloy Anode Toward High-Performance Sulfide-Based All-Solid-State Lithium Batteries. *Adv. Mater.* **2024**, 36, 2407128.

[9] T. Yang; W. Zhang; Y. Liu; J. Zheng; Y. Xia; X. Tao; Y. Wang; X. Xia; H. Huang; Y. Gan; X. He; J. Zhang. High-Performance Solid Lithium Metal Batteries Enabled by LiF/LiCl/LiIn Hybrid SEI via InCl_3_-Driven In Situ Polymerization of 1,3-Dioxolane. *Small* **2023**, 19, 2303210.

[10] W. Li; J. Gao; H. Tian; X. Li; S. He; J. Li; W. Wang; L. Li; H. Li; J. Qiu; W. Zhou. SnF_2_-Catalyzed Formation of Polymerized Dioxolane as Solid Electrolyte and Its Thermal Decomposition Behavior. *Angew. Chem., Int. Ed.* **2022**, 61, e202114805.

[11] H. Yang; B. Zhang; M. Jing; X. Shen; L. Wang; H. Xu; X. Yan; X. He. In Situ Catalytic Polymerization of a Highly Homogeneous PDOL Composite Electrolyte for Long-Cycle High-Voltage Solid-State Lithium Batteries. *Adv. Energy Mater.* **2022**, 12, 2201762.

[12] Y. So; H. Seo; S. H. Lee; E. Lee; J. Lee; J. Kang; Y. Y. Kim; B. H. Kim; S. Mhin. Enhanced Electrochemical Performance of Aqueous Zn-Ion Batteries Based on Na_2_V_6_O_16_·2H_2_O Cathodes: Insights from DFT and Synchrotron X-ray Analysis. *J. Mater. Chem. A* **2025**, 13, 8761–8773.

[13] P. Xu; Y. C. Gao; Y. X. Huang; Z. Y. Shuang; W. J. Kong; X. Y. Huang; W. Z. Huang; N. Yao; X. Chen; H. Yuan; C. Z. Zhao; J. Q. Huang; Q. Zhang. Weakly Solvating Electrolyte: Regulating Polysulfide Intermediates for High-Performance Lithium–Sulfurized Polyacrylonitrile Batteries. *Adv. Mater.* **2024**, 36, 2409489.

[14] D. Chen; M. Zhu; P. Kang; T. Zhu; H. Yuan; J. Lan; X. Yang; G. Sui. Self-Enhancing Gel Polymer Electrolyte by In Situ Construction for Enabling Safe Lithium Metal Battery. *Adv. Sci.* **2022**, 16, 2103663.

[15] B. H. Zhang; W. X. Wen; H. Y. Wang; Y. L. Hou; J. Z. Chen; D. L. Zhao. In Situ Generated Hybrid Interface in Poly(1,3-dioxolane) Quasi-Solid Electrolyte and Extended Sulfone Cosolvent for Lithium-Metal Batteries. *Chem. Eng. J.* **2023**, 472, 144990.

[16] D. Wang, B. Jin, J. Huang, X. Yao, Y. Ren, X. Xu, X. Han, F. Li, X. Zhan, Q. Zhang. Laponite-Supported Gel Polymer Electrolyte with Multiple Lithium-Ion Transport Channels for Stable Lithium Metal Batteries. *ACS Appl. Mater. Interfaces* 2023, ***15***, 32385.

[17] S. Wang, L. Zhou, M. K. Tufail, L. Yang, P. Zhai, R. Chen, W. Yang. In-Situ Synthesized Non-flammable Gel Polymer Electrolyte Enable Highly Safe and Dendrite-Free Lithium Metal Batteries. *Chem. Eng. J.* 2021, ***415***, 128846.

[18] T. Zhu, G. Wang, J. Hou, W. Sun, C. Song, Q. Yuan, C. Zhang, X. Lei, Y. Su, M. Chen, Y. Song, J. Zhao. Heterogeneous Engineered Solid Electrolyte for Seamless and Stable Integration of Anode and Cathode. *Adv. Funct. Mater.* 2025, ***35***, 2501870.

[19] S. Zheng, Y. Chen, K. Chen, S. Yang, R. Bagherzadeh, Y. E. Miao, T. Liu. In situ construction of polyether-based composite electrolyte with bi-phase ion conductivity and stable electrolyte/electrode interphase for solid-state lithium metal batteries. *J. Mater. Chem. A* 2022, ***10***, 19641.
